# Patient-Level Discordance Between SCORE2 Cardiovascular Risk and PD-1/PD-L1 Checkpoint-Expression Profiles in People Living with HIV: A Secondary Cohort Analysis

**DOI:** 10.3390/pathogens15080871

**Published:** 2026-08-20

**Authors:** Bogusz Aksak-Wąs, Karolina Skonieczna-Żydecka, Danuta Cembrowska-Lech, Miłosz Parczewski, Filip Lewandowski, Karol Serwin, Kaja Mielczak, Mateusz Bruss, Franciszek Lenkiewicz, Milena Rafalska-Kosior, Laura Lesiewska, Paulina Niedźwiedzka-Rystwej

**Affiliations:** 1 Department of Infectious, Tropical Diseases and Acquired Immunodeficiencies, Pomeranian Medical University in Szczecin, 70-204 Szczecin, Poland; 2 Department of Biochemical Science, Pomeranian Medical University in Szczecin, 70-204 Szczecin, Poland; 3 Institute of Biology, University of Szczecin, 70-453 Szczecin, Polandpaulina.niedzwiedzka-rystwej@usz.edu.pl (P.N.-R.); 4 Center for Experimental Immunology and Immunobiology in Infectious Diseases and Cancer, University of Szczecin, 70-453 Szczecin, Poland; 5 Regional Center for Digital Medicine, Department of Genomics and Forensic Genetics, Faculty of Medicine, Pomeranian Medical University in Szczecin, 70-204 Szczecin, Poland

**Keywords:** HIV, SCORE2, cardiovascular risk, immune exhaustion, PD-1, PD-L1, smoking, CD4+/CD8+ ratio, immune checkpoints, antiretroviral therapy

## Abstract

Background: Conventional cardiovascular risk scores do not directly characterize immune checkpoint-expression phenotypes in people with HIV. We examined patient-level alignment between SCORE2 and PD-1/PD-L1 checkpoint expression in a secondary analysis of a previously characterized parent cohort. Methods: Data from 100 PWH followed at a single HIV center were analyzed. SCORE2, lipid parameters, systolic blood pressure, CD4+/CD8+ ratio, and flow-cytometric PD-1/PD-L1 expression on T-cell, B-cell, and NK-cell subsets were assessed at baseline and after 12 months. Composite immune-exhaustion indices were derived from standardized checkpoint-marker values. Discordance profiles were defined using quartile thresholds for endpoint SCORE2 and immune exhaustion. Results: Smokers had higher endpoint SCORE2 than nonsmokers (median 3.5% vs. 1.0%; *p* = 0.001), but smoking was not associated with higher composite or individual checkpoint-defined exhaustion. Endpoint SCORE2 correlated with classical cardiovascular variables but not with the composite exhaustion index (ρ = 0.075; *p* = 0.468) or individual PD-1/PD-L1 markers. Discordant cardiovascular–immune profiles were observed in 10 of 96 evaluable participants (10.4%). Conclusions: SCORE2 and PD-1/PD-L1 checkpoint-expression profiles showed limited contemporaneous alignment in this cohort. The identified discordant profiles are exploratory and require external validation.

## 1. Introduction

The introduction of effective combination antiretroviral therapy has transformed human immunodeficiency virus (HIV) infection from a rapidly progressive and frequently fatal disease into a chronic, clinically manageable condition. However, durable virological suppression does not fully normalize immune function or eliminate the excess burden of non-acquired immunodeficiency syndrome (AIDS) comorbidities. In people with HIV (PWH), long-term prognosis remains shaped not only by HIV ribonucleic acid (RNA) suppression, but also by the extent and quality of immune restoration. Previous work from Polish HIV cohorts has shown that immune restoration is clinically meaningful and associated with long-term survival, while the probability of achieving satisfactory CD4+ T-cell recovery depends on baseline clinical status, late presentation, and host-related immunogenetic factors [1,2,3,4,5]. These observations support a broader view of HIV care, in which virological control is necessary but insufficient to define biological recovery.

Cardiovascular disease has become one of the most important non-AIDS comorbidities in PWH. Classical cardiovascular risk prediction in Europe is commonly based on Systematic Coronary Risk Evaluation 2 (SCORE2), which estimates the 10-year risk of fatal and non-fatal cardiovascular events in apparently healthy individuals aged 40–69 years [6]. HIV-specific cardiovascular risk equations, including models derived from the Data Collection on Adverse Events of Anti-HIV Drugs (D:A:D) cohort, have also been developed to incorporate HIV-related and treatment-related variables [7]. Nevertheless, the performance of cardiovascular risk scores in PWH remains imperfect. A systematic review and meta-analysis showed that several commonly used models tend to miscalibrate or underpredict cardiovascular risk in this population [8]. The clinical relevance of this problem was further underscored by the study “Evaluating the Use of Pitavastatin to Reduce the Risk of Cardiovascular Disease in HIV-Infected Adults (REPRIEVE)”, which demonstrated that pitavastatin reduced major adverse cardiovascular events among PWH receiving antiretroviral therapy, even though many participants had low-to-moderate predicted cardiovascular risk by conventional criteria [9]. More recent analyses from REPRIEVE again emphasized that both general-population and HIV-specific risk equations may perform inconsistently across sex, race, and geographic strata, highlighting the need for more precise risk stratification in contemporary HIV populations [10].

The cardiovascular phenotype of PWH is particularly complex because conventional risk factors coexist with HIV-related immune perturbation, chronic inflammation, antiretroviral exposure, metabolic changes, and coinfections. In a young central/eastern European cohort of PWH with suppressed viremia, subclinical cardiovascular abnormalities were detectable despite the absence of advanced age or overt clinical cardiovascular disease [11]. Echocardiographic analyses in virally suppressed PWH have also suggested that cardiac structural and functional changes may occur even in clinically stable patients [12]. Together, these observations indicate that conventional cardiovascular risk assessment captures only one part of the cardiovascular vulnerability observed in PWH.

Smoking remains one of the strongest modifiable determinants of cardiovascular morbidity and mortality in HIV care. In a nationwide population-based cohort, mortality attributable to smoking among HIV-infected individuals was substantial, and the loss of life-years related to smoking exceeded the loss attributable to HIV itself in some strata [13]. Data from the D:A:D study also showed that cardiovascular disease risk decreases after smoking cessation in PWH, supporting smoking cessation as a central preventive intervention [14]. Because smoking is directly incorporated into SCORE2 and related risk algorithms, smokers are expected to be classified as having higher conventional cardiovascular risk. However, whether smoking-related cardiovascular risk is paralleled by a comparable increase in checkpoint-defined immune exhaustion is less certain.

Persistent immune activation and inflammation provide a plausible biological bridge between HIV infection and cardiovascular disease. In treated and untreated HIV infection, inflammatory and coagulation biomarkers such as interleukin-6, high-sensitivity C-reactive protein, and D-dimer have been associated with cardiovascular events and mortality [15]. Immunological indices such as the CD4+/CD8+ ratio have also been proposed as markers of immune senescence, residual immune dysfunction, and risk of serious non-AIDS events [16,17,18]. These data suggest that cardiovascular vulnerability in PWH may be only partly represented by classical clinical variables, while additional immune-defined dimensions may contribute to the broader risk phenotype.

Among immune regulatory pathways, programmed cell death protein 1 (PD-1) and its ligand (PD-L1) occupy a central position in the biology of T-cell dysfunction. Seminal studies in HIV infection demonstrated that PD-1 is upregulated on HIV-specific CD8+ T cells, correlates with viral load and impaired effector function, and is inversely related to CD4+ T-cell counts [19,20]. PD-1 expression may decrease after antiretroviral therapy, but checkpoint abnormalities can persist despite virological suppression [21]. Moreover, CD4+ T cells expressing immune checkpoint receptors, including PD-1, TIGIT, and LAG-3, have been shown to contribute to HIV persistence during antiretroviral therapy [22]. These findings establish PD-1/PD-L1 not merely as phenotypic markers, but as biologically relevant components of chronic immune dysregulation in HIV infection.

Recent studies further support the clinical relevance of PD-1/PD-L1 profiling in infection-related immune dysregulation. In Coronavirus Disease 2019 (COVID)-19, PD-1/PD-L1 expression was associated with disease severity and was proposed as a marker of immune perturbation [23]. In PWH, persistent PD-1/PD-L1 expression was observed despite virological suppression and CD4+ T-cell recovery, suggesting that immunological recovery and checkpoint-defined exhaustion may represent overlapping but non-identical biological states [24]. Additional analyses showed that PD-1/PD-L1 markers were only weakly associated with conventional immune-reconstitution metrics during antiretroviral therapy [25]. Furthermore, antiretroviral regimen class may influence immune checkpoint phenotypes, with non-integrase strand transfer inhibitor-based regimens showing potentially more favorable associations with selected immune-exhaustion markers in one cohort analysis [26]. Our previous group-level analysis in reference [24] already showed that higher PD-1/PD-L1 expression in newly diagnosed participants was not accompanied by a higher between-group SCORE2 value. We therefore treat this observation as the premise for the present study rather than as a novel finding. The distinct objective of the present secondary analysis was to quantify contemporaneous SCORE2–checkpoint alignment within individual participants and to identify direction-specific discordant profiles. All analytic samples reported in references [24,25,26] are nested within the present parent cohort. These findings raise the possibility that checkpoint-defined immune risk may not be adequately inferred from CD4+ T-cell count, CD4+/CD8+ ratio, virological suppression, or classical cardiovascular risk scores alone.

Despite the recognized excess cardiovascular burden in PWH and the growing interest in immune checkpoint biology, it remains unclear whether SCORE2-defined cardiovascular risk aligns with PD-1/PD-L1-defined immune exhaustion at the individual patient level. This question is clinically relevant because concordant and discordant phenotypes may have different implications. Patients with high SCORE2 and high immune exhaustion may represent a subgroup with both conventional and immune-defined risk accumulation. Conversely, patients with high SCORE2 but low checkpoint-defined exhaustion, or low SCORE2 but high checkpoint-defined exhaustion, may be misclassified if risk assessment is restricted to only one biological axis. The identification of such discordant profiles could support a more nuanced approach to cardiovascular and immunological monitoring in PWH.

In this study, we investigated the relationship between SCORE2-defined cardiovascular risk and checkpoint-defined immune exhaustion in PWH. We assessed whether smoking, as a major conventional cardiovascular risk factor, was associated with parallel changes in PD-1/PD-L1 expression on T-cell, B-cell, and natural killer (NK)-cell compartments. We further examined correlations between SCORE2, classical cardiovascular parameters, immune-reconstitution indices, and checkpoint markers, and constructed patient-level discordance profiles based on cardiovascular risk and composite immune-exhaustion indices. We hypothesized that endpoint SCORE2 and checkpoint-expression profiles would show limited alignment at the individual-participant level.

## 2. Materials and Methods

Longitudinal data were collected from 100 people living with HIV followed at the Department of Infectious, Tropical Diseases and Acquired Immunodeficiencies, Pomeranian Medical University in Szczecin, Poland. The study protocol was approved by the Bioethical Committee of the Pomeranian Medical University in Szczecin, Poland (approval number KB-006/40/2023). Written informed consent was obtained from all participants, and all data were pseudonymized before statistical analysis. The cohort included both newly diagnosed and long-term-treated people with HIV. Newly diagnosed patients were defined as individuals entering care after a recent HIV diagnosis and not previously receiving antiretroviral therapy. Long-term-treated patients were defined as individuals already receiving antiretroviral therapy for at least 24 months and remaining under specialist HIV care. In newly diagnosed patients, antiretroviral therapy was initiated according to the standard of care within no more than two weeks from the first presentation to the clinic; most were initiated at the first visit in the clinic. Participants were included in individual analyses according to the availability of the required clinical, laboratory, cardiovascular, and immunophenotypic variables. The relationship between the present parent cohort and our previous publications was assessed at the patient level using pseudonymized study identifiers. Reference [1] was based on an entirely separate historical cohort, and no patient-level identifiers matched between the datasets; the overlap was therefore 0/100 participants in the current cohort. All participants included in the analytic samples of references [24,25,26] are present in the current dataset, corresponding to 79/79, 52/52, and 58/58 participants, respectively. Using the current 100-participant cohort as the denominator, the corresponding overlaps are 79/100, 52/100, and 58/100. Because the 52 participants in reference [25] are nested within both references [24,26], while reference [24] additionally includes 27 long-term-treated participants, the union of participants represented in references [24,25,26] is 85/100. Fifteen long-term-treated participants in the present cohort were not included in any of these previous analytic samples.

A single harmonized dataset was analyzed. It included sociodemographic variables, including age, sex, smoking status, and country of origin; HIV-related variables, including HIV subtype, clinical group, antiretroviral therapy category, and active hepatitis C virus (HCV) status; standard immunological indices, including CD4+ and CD8+ T-cell counts and CD4+/CD8+ ratio at baseline and after 12 months (endpoint of the analysis); metabolic and cardiovascular parameters, including lipid profile, systolic blood pressure, and SCORE2 at baseline and endpoint; and flow-cytometric lymphocyte phenotyping with assessment of PD-1 and PD-L1 expression at two time points.

SCORE2 was calculated using standard cardiovascular risk variables, including age, sex, smoking status, systolic blood pressure, total cholesterol, and High-Density Lipoprotein (HDL) cholesterol. Endpoint SCORE2 was used as the main measure of classical cardiovascular risk. Because SCORE2 is conventionally intended for apparently healthy individuals aged 40–69 years, the age distribution of the cohort and the number of participants outside this interval were evaluated. SCORE2 values outside the 40–69-year interval were retained as exploratory conventional risk-score descriptors, and age-restricted sensitivity analysis was performed among participants aged 40–69 years.

Flow-cytometric immunophenotyping included assessment of PD-1 and PD-L1 expression on selected lymphocyte subsets, including CD3+ T cells, CD4+ T cells, CD19+ B cells, and NK cells where available. Checkpoint-marker expression was analyzed as the percentage of PD-1- or PD-L1-positive cells within the respective lymphocyte subset.

Checkpoint-defined immune exhaustion was assessed using individual PD-1/PD-L1 markers and a composite immune-exhaustion index. The primary composite index was calculated as the mean of standardized endpoint T-cell and B-cell checkpoint markers, including PD-1 and PD-L1 expression on CD3+, CD4+, and CD19+ lymphocyte subsets. Each marker was transformed into a z-score before averaging. An expanded secondary index additionally included endpoint NK-cell PD-L1 expression.

Antiretroviral treatment (ART) was categorized according to the presence of an integrase strand transfer inhibitor and the use of tenofovir alafenamide or tenofovir disoproxil fumarate. The following analytical categories were created: integrase strand transfer inhibitor (INSTI) + tenofovir alafenamide (TAF), INSTI + tenofovir disoproxil fumarate (TDF), INSTI without tenofovir, TAF without INSTI, and non-INSTI/non-TAF regimens. In sensitivity analyses, a simplified classification comparing INSTI-based and non-INSTI-based regimens was also used.

### 2.1. Statistical Analysis

All statistical analyses were performed using Python v3.14.5 (https://www.python.org/; Release date: 10 May 2026; accessed on 10 August 2026) in PyCharm Professional integrated development environment (v2026.1.2) (JetBrains s.r.o.; https://www.jetbrains.com/pycharm/; Release date: 15 May 2026; accessed on 10 August 2026) for Linux (Fedora v44) (https://fedoraproject.org/; Release date: 28 April 2026; accessed on 10 August 2026).

Continuous variables were summarized using medians and interquartile ranges (IQRs), and categorical variables were summarized as counts and percentages. All analyses were two-sided. Nominal statistical significance was defined as *p* < 0.05. Where multiple immune markers or correlation panels were evaluated simultaneously, Benjamini–Hochberg false-discovery-rate (FDR) correction was applied, and FDR-adjusted q-values were reported.

Missingness was assessed for all variables used in the analyses. No imputation was performed. Participants were included in each analysis according to complete-case availability for the variables required for that analysis, and analysis-specific sample sizes were reported in the Supplementary Tables S2 and S3, respectively.

Endpoint immune exhaustion was evaluated using both individual cytometric markers and predefined composite indices. The primary composite exhaustion index was calculated as the mean of standardized endpoint T- and B-cell checkpoint markers, including PD-1 and PD-L1 expression on CD3+, CD4+, and CD19+ lymphocyte subsets. Each component marker was transformed to a z-score before averaging, so that markers measured on different percentage scales contributed comparably. An expanded secondary exhaustion index additionally included endpoint NK-cell PD-L1 expression.

Comparisons between smokers and nonsmokers were performed separately for classical cardiovascular variables and immune-exhaustion variables. Continuous variables were compared using the Mann–Whitney U test because several immunological and biochemical variables showed skewed distributions and because subgroup sizes were modest. Effect sizes for continuous comparisons were reported using Cliff’s delta. Categorical variables were compared using Fisher’s exact test.

To assess whether SCORE2-defined cardiovascular risk was aligned with endpoint checkpoint-defined immune exhaustion, Spearman rank correlations were calculated between endpoint SCORE2 and the composite exhaustion indices, individual PD-1/PD-L1 markers, NK-cell parameters, CD4+/CD8+ ratio, lipid parameters, and systolic blood pressure.

Adjusted associations between smoking and immune-exhaustion outcomes were evaluated using ordinary least-squares linear regression models with heteroscedasticity-consistent type 3 (HC3) robust standard errors. The main adjusted models included smoking status as the exposure and immune-exhaustion outcomes as dependent variables. Outcomes included the T/B-cell composite exhaustion index, the expanded T/B/NK exhaustion index, and individual endpoint checkpoint markers. Covariates were selected a priori and included age, sex, clinical group, antiretroviral therapy category, HIV subtype category, and active HCV status when available.

For adjusted regression models, potential influence of individual observations was assessed using leverage values and Cook’s distance. High leverage was defined as leverage greater than 2p/n, where *p* is the number of model parameters and *n* is the model sample size. Potentially influential observations were defined as Cook’s distance greater than 4/n, and sensitivity models were repeated after excluding observations above this threshold.

To evaluate patient-level discordance between classical cardiovascular risk and checkpoint-defined immune exhaustion, predefined discordance profiles were constructed using endpoint SCORE2 and the primary T/B-cell composite exhaustion index. In the primary discordance analysis, high cardiovascular risk was defined as SCORE2 in the upper quartile, low cardiovascular risk as SCORE2 in the lower quartile, high immune exhaustion as composite exhaustion index in the upper quartile, and low immune exhaustion as composite exhaustion index in the lower quartile. Patients were classified into clinically interpretable profiles: high cardiovascular risk/high exhaustion, high cardiovascular risk/low exhaustion, low cardiovascular risk/high exhaustion, low cardiovascular risk/low exhaustion, or intermediate/unclassified. A secondary discordance definition used median splits for SCORE2 and the composite exhaustion index to provide a less stringent but more inclusive sensitivity classification. Discordance counts were summarized overall and stratified by smoking status, clinical group, and active HCV status.

Additional robustness analyses were performed to evaluate the stability of the main discordance model. These included analyses using the T/B-cell exhaustion index only, analyses using the expanded T/B/NK exhaustion index, quartile-defined discordance, median-defined discordance, complete-case restriction, exclusion of active HCV, newly diagnosed-only subgroup analysis, long-term-treated-only subgroup analysis, alternative ART classification as INSTI-based versus non-INSTI-based therapy, and winsorized immune-marker analysis to reduce the influence of extreme checkpoint-expression values.

For the winsorized sensitivity analysis, each endpoint checkpoint-expression marker was capped at the fifth and 95th percentiles of its observed distribution before z-score standardization and construction of the composite immune-exhaustion indices.

For the boxplot, observations outside the 1.5 × IQR whisker range were identified but were not automatically excluded. Extreme values were reviewed for biological and measurement plausibility, including confirmation that flow-cytometric percentage values remained within the possible 0–100% range.

### 2.2. Immunophenotyping

**Figure 1:**
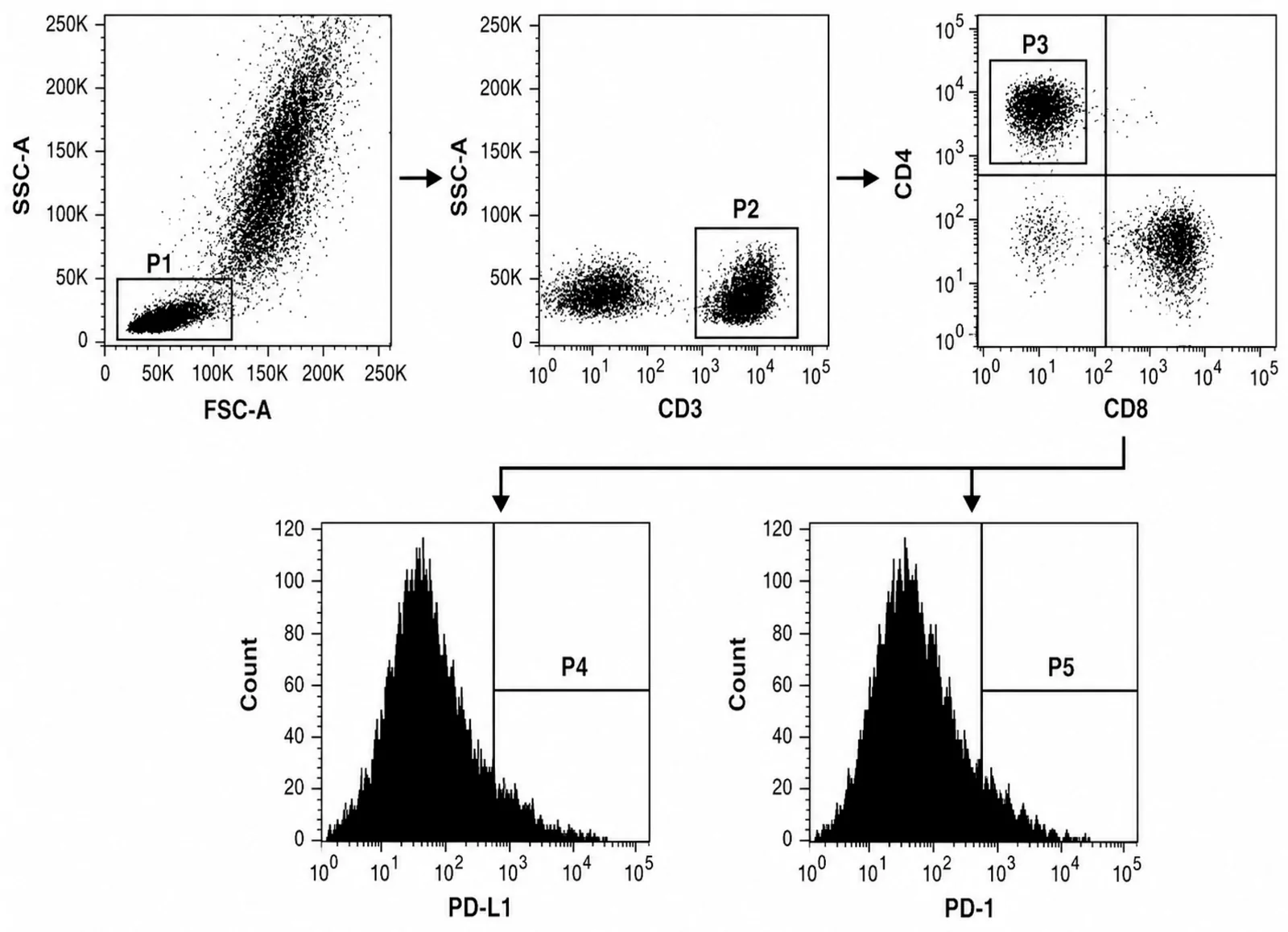
Flow cytometry gating strategy for the analysis of PD-1 and PD-L1 expression in CD3+CD4+ T cells. The lymphocyte population was initially identified based on FSC-A and SSC-A characteristics. CD3+ T lymphocytes were subsequently selected and further subdivided according to CD4 and CD8 expression. CD3+CD4+ T cells were gated for downstream analysis of PD-L1 and PD-1 expression using histogram-based analysis. Rectangular regions labelled P1–P5 denote sequential analysis gates, and arrows indicate the order of gating. Vertical solid lines in the histogram panels define the thresholds for PD-L1- and PD-1-positive events. Fluorescence-intensity axes are presented on a logarithmic scale (10^0^–10^5^). FSC-A, forward-scatter area; SSC-A, side-scatter area.

**Figure 2:**
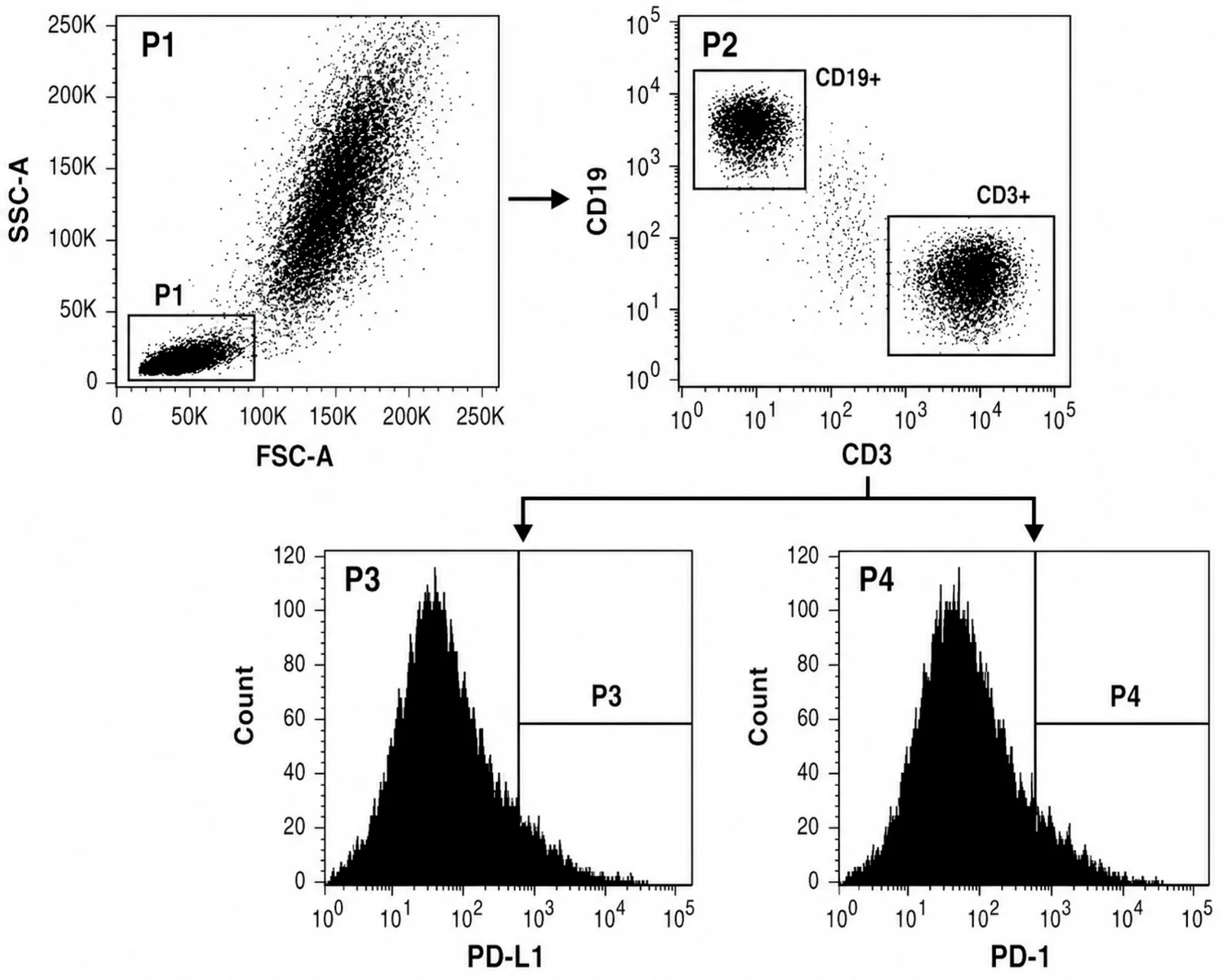
Flow cytometry gating strategy for the analysis of PD-1 and PD-L1 expression in T and B lymphocytes. Following identification of the lymphocyte population based on FSC-A and SSC-A characteristics, T and B lymphocytes were distinguished according to CD3+ and CD19+ expression. CD3+ T cells and CD19+ B cells were gated separately, and PD-L1 and PD-1 expression was subsequently assessed within the respective cell populations using histogram-based analysis. Rectangular regions labelled P1–P4 denote sequential analysis gates, and arrows indicate the order of gating. Vertical solid lines in the histogram panels define the thresholds for PD-L1- and PD-1-positive events. Fluorescence-intensity axes are presented on a logarithmic scale (10^0^–10^5^). FSC-A, forward-scatter area; SSC-A, side-scatter area.

**Figure 3:**
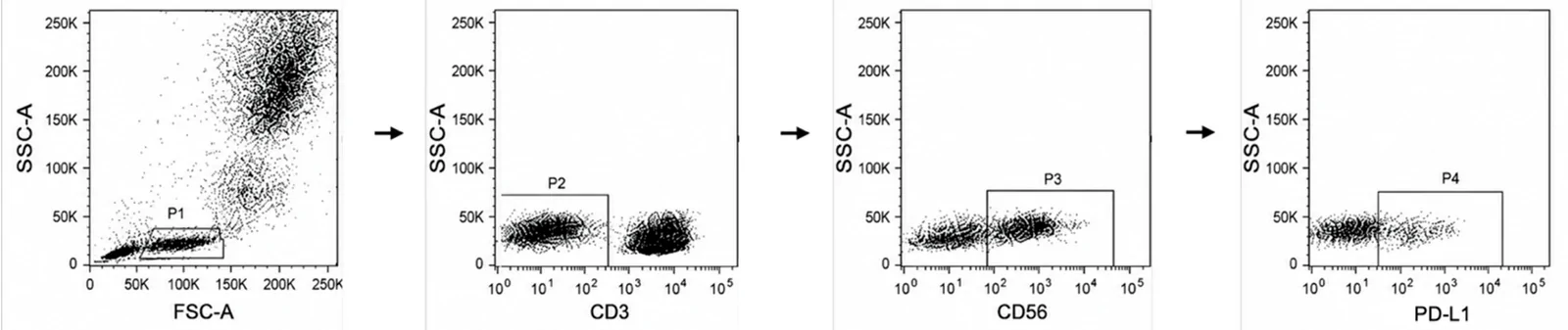
Flow cytometry gating strategy for the analysis of PD-1 and PD-L1 expression in NK cells. Following identification of the lymphocyte population based on FSC-A and SSC-A characteristics, CD3^−^ cells were selected. NK cells were subsequently identified as CD3−CD56+ cells, and PD-L1 expression was assessed within the gated NK-cell population. Gates P1–P4 are displayed from left to right in sequential order, and the vertical line in the final panel defines PD-L1-positive events. Fluorescence-intensity axes are presented on a logarithmic scale (10^0^–10^5^). FSC-A, forward-scatter area; SSC-A, side-scatter area.

Peripheral blood samples were collected into ethylenediaminetetraacetic acid (EDTA)-containing tubes and processed immediately after collection. Flow-cytometry variables analyzed in this secondary study were generated within the parent protocol using the laboratory workflow previously reported in references [24,25,26]. Briefly, freshly collected EDTA-anticoagulated whole blood was stained with fluorochrome-conjugated monoclonal antibodies, erythrocytes were lysed, and washed cells were acquired on an eight-color FACSCanto II flow cytometer (BD Biosciences; Franklin Lakes, NJ, USA), with 10,000–50,000 events recorded per sample. After forward scatter (FSC) and side scatter (SSC) lymphocyte gating and, where applicable, doublet exclusion, CD3+ T cells, CD3+CD4+ T cells, CD19+ B cells, and CD3−CD56+ NK cells were identified. Unstained controls were used to assess background fluorescence; marker-positive populations were defined using the predefined gating thresholds applied consistently across all samples. The present secondary analysis used the previously generated percentage-positive variables and did not involve sample re-gating. Full antibody-panel and T- and B-cell gating details are provided in references [24,25,26]. Representative sequential gating strategies for CD3+CD4+ T cells, T and B lymphocytes, and NK cells are presented in Figure 1, Figure 2 and Figure 3, respectively. Representative sequential gating strategies for CD3+CD4+ T cells, T and B lymphocytes and NK cells are presented in Figure 1, Figure 2 and Figure 3, respectively.

## 3. Results

### 3.1. Cohort Characteristics

The study included 100 people living with HIV, with a median age of 41.5 years (IQR: 34.8–47.0); 56/100 participants (56.0%) were male and 54/100 (54.0%) were smokers (Supplementary Table S1). The cohort included 58/100 newly diagnosed participants (58.0%) and 42/100 long-term-treated participants (42.0%). The most common ART categories were INSTI + TAF (51/99, 51.5%) and INSTI + TDF (37/99, 37.4%). Among the full cohort, subtype A6 was recorded in 44/100 (44.0%) and subtype B in 22/100 (22.0%) of participants. Active HCV RNA was present in 12/100 participants (12.0%).

At endpoint, the median CD4+ T-cell count was 603.5 cells/µL (IQR: 415.2–834.5), the median CD8+ T-cell count was 741.5 cells/µL (IQR: 531.2–990.5), and the median CD4+/CD8+ ratio was 0.86 (IQR: 0.48–1.25). The median endpoint SCORE2 was 2.0% (IQR: 0.0–4.0), with median systolic blood pressure of 131.5 mmHg (IQR: 125.0–147.0). Lipid parameters were within a broad clinical range, with median LDL cholesterol of 123.0 mg/dL (IQR: 93.0–139.0), HDL cholesterol of 50.3 mg/dL (IQR: 42.3–59.4), triglycerides of 98.0 mg/dL (IQR: 75.0–152.2), and total cholesterol of 175.0 mg/dL (IQR: 150.0–202.0). The median T/B-cell exhaustion index was −0.170 (IQR: −0.394 to 0.303), and the expanded T/B/NK exhaustion index was −0.184 (IQR: −0.426 to 0.369).

### 3.2. Smoking Was Associated with Higher SCORE2-Defined Cardiovascular Risk

Smoking status was associated with a clear shift in SCORE2-defined cardiovascular risk. At endpoint, smokers had a higher SCORE2 than nonsmokers, with median values of 3.5% (IQR: 1.0–7.0%) and 1.0% (IQR: 0.0–2.0%), respectively (Mann–Whitney U test, *p* = 0.001; FDR-adjusted q < 0.05). A similar difference was already present at baseline, where median SCORE2 was 3.0% (IQR: 1.0–7.0%) in smokers and 1.0% (IQR: 0.0–2.0%) in nonsmokers (*p* = 0.001; FDR-adjusted q < 0.05). The effect size for the smoker–nonsmoker contrast was moderate for both endpoint SCORE2 and baseline SCORE2, consistent with higher calculated cardiovascular risk in smokers.

**Figure 4:**
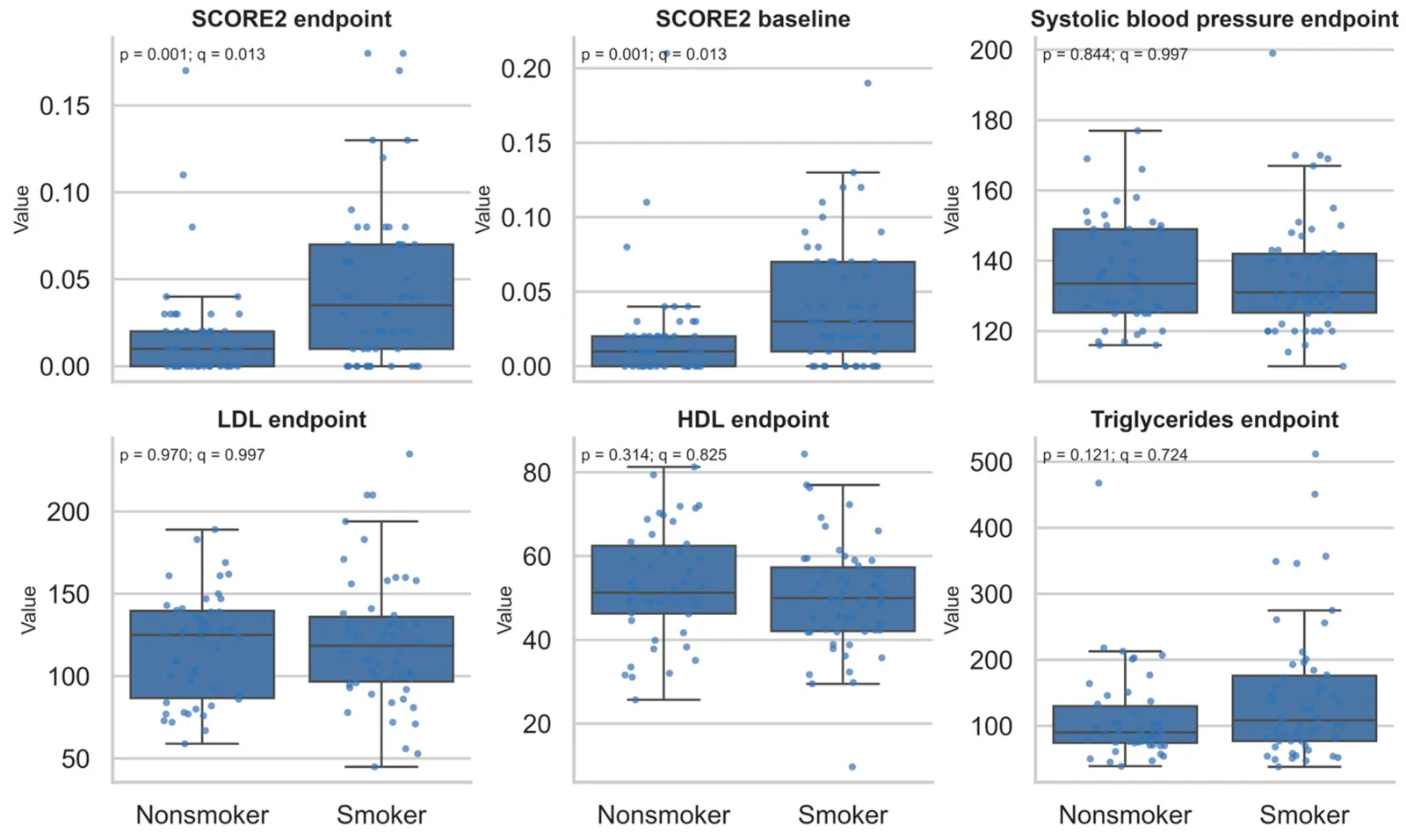
Distribution of SCORE2 and classical cardiovascular-risk parameters according to smoking status. Continuous variables were compared using two-sided Mann–Whitney U tests, with Benjamini–Hochberg false-discovery-rate correction applied across the displayed cardiovascular variables. Boxes represent the interquartile range, central lines indicate medians, and whiskers extend to the most extreme observations within 1.5 × IQR of the lower and upper quartiles. Overlaid dots represent individual participants; observations beyond the whiskers were retained in the analyses. SCORE2 is displayed as a proportion, whereas systolic blood pressure and lipid concentrations are expressed in mmHg and mg/dL, respectively.

By contrast, individual classical cardiovascular-risk components and lipid parameters did not show comparable group-level separation. Endpoint systolic blood pressure was similar in smokers and nonsmokers (median 131 mmHg [IQR: 125–142] vs. 134 mmHg [IQR: 125–149], respectively; *p* = 0.844). LDL cholesterol also did not differ between groups (118.5 mg/dL [IQR: 96.8–136.0] vs. 125.0 mg/dL [IQR: 86.8–139.8]; *p* = 0.970), nor did HDL cholesterol (50.0 mg/dL [IQR: 42.1–57.3] vs. 51.3 mg/dL [IQR: 46.3–62.5]; *p* = 0.314). Triglycerides were numerically higher among smokers (108.5 mg/dL [IQR: 77.0–176.0]) than nonsmokers (90.0 mg/dL [IQR: 74.3–130.0]), but this difference did not remain statistically significant (*p* = 0.121; FDR-adjusted q > 0.05). These findings indicate that smoking status primarily separated patients by calculated SCORE2 risk, rather than by a broad shift across all measured lipid or blood-pressure variables. The distributions of SCORE2 and the individual classical cardiovascular-risk parameters according to smoking status are shown in Figure 4.

### 3.3. Smoking Was Not Associated with a Consistent Increase in Checkpoint-Defined Immune Exhaustion

The composite immune-exhaustion index was numerically lower in smokers than nonsmokers, but the difference was not statistically significant (median −0.272 [IQR: −0.426 to 0.313] vs. −0.0065 [IQR: −0.420 to 0.445], respectively; Mann–Whitney U test, *p* = 0.373; FDR-adjusted q > 0.05). Similarly, CD3+ T-cell PD-1 expression was numerically lower in smokers (22.3% [IQR: 19.0–28.0]) than nonsmokers (25.2% [IQR: 20.1–35.3]), but this comparison did not reach statistical significance after correction for multiple testing (*p* = 0.154; FDR-adjusted q > 0.05). CD4+ T-cell PD-1 expression showed a similar pattern, with median values of 20.1% (IQR: 13.8–27.2) in smokers and 21.5% (IQR: 16.7–29.6) in nonsmokers (*p* = 0.241; FDR-adjusted q > 0.05).

**Figure 5:**
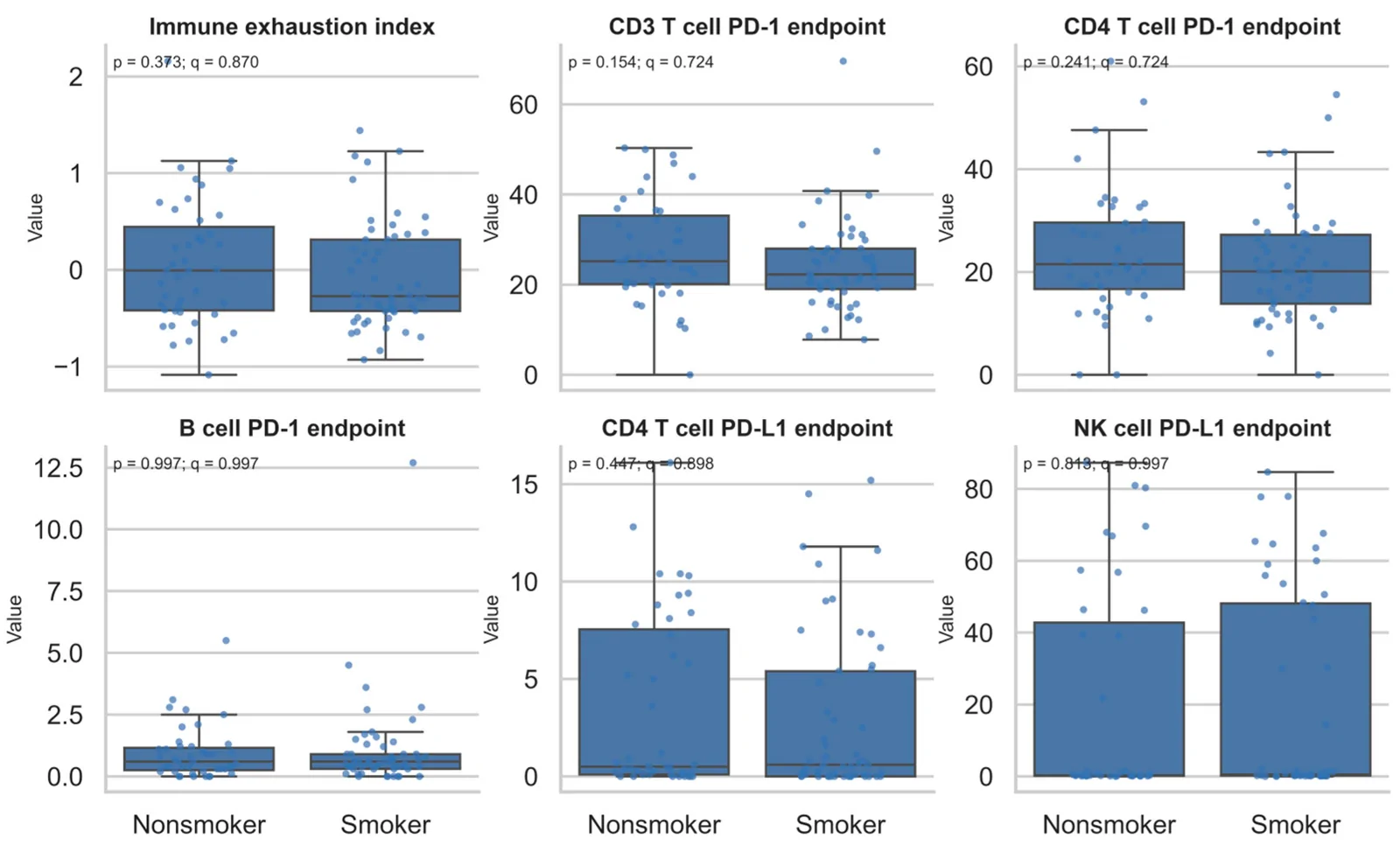
Distribution of checkpoint-defined immune-exhaustion markers according to smoking status. Box-and-whisker plots compare smokers and nonsmokers for the composite immune-exhaustion index and selected endpoint PD-1/PD-L1 markers measured on T-cell, B-cell, and NK-cell compartments. The immune-exhaustion index was derived from standardized endpoint checkpoint-marker values. Group comparisons were performed using two-sided Mann–Whitney U tests, with Benjamini–Hochberg false-discovery-rate correction applied across the immune-marker panel. Boxes represent the interquartile range, central lines indicate medians, and whiskers extend to the most extreme observations within 1.5 × IQR of the lower and upper quartiles. Overlaid dots represent individual participants; observations beyond the whiskers were retained. Checkpoint-marker expression is presented as the percentage of marker-positive cells, whereas the composite exhaustion index is dimensionless.

Other checkpoint markers also did not show evidence of a smoking-related shift. B-cell PD-1 expression was nearly identical in the two groups (median 0.6% in both smokers and nonsmokers; *p* = 0.997). CD4+ T-cell PD-L1 expression was likewise similar between smokers and nonsmokers (0.6% [IQR: 0.0–5.4] vs. 0.5% [IQR: 0.1–7.6], respectively; *p* = 0.447). NK-cell PD-L1 expression showed high interindividual variability in both groups and did not differ significantly by smoking status (0.55% [IQR: 0.20–48.13] in smokers vs. 0.30% [IQR: 0.15–42.80] in nonsmokers; *p* = 0.813). To sum up, although smoking was associated with higher SCORE2-defined cardiovascular risk, this was not accompanied by a parallel increase in endpoint PD-1/PD-L1-defined immune exhaustion. The corresponding distributions of the composite index and individual checkpoint markers are shown in Figure 5.

### 3.4. SCORE2 Showed Low or Absent Correlations with Endpoint PD-1/PD-L1 Expression

Endpoint SCORE2 correlated positively with systolic blood pressure (Spearman ρ = 0.414, *p* < 0.001, q < 0.001), total cholesterol (ρ = 0.440, *p* < 0.001, q < 0.001), LDL cholesterol (ρ = 0.398, *p* < 0.001, q < 0.001), non-HDL cholesterol (ρ = 0.482, *p* < 0.001, q < 0.001), and triglycerides (ρ = 0.281, *p* = 0.0047, q = 0.0147). HDL cholesterol was not significantly correlated with SCORE2 (ρ = −0.048, *p* = 0.635, q = 0.760), and the endpoint CD4/CD8 ratio showed only a weak, non-significant inverse association (ρ = −0.122, *p* = 0.233, q = 0.407).

In contrast, endpoint SCORE2 showed no meaningful correlation with composite or individual immune-exhaustion measures. The correlation between SCORE2 and the composite immune-exhaustion index was low and non-significant (ρ = 0.075, *p* = 0.468, q = 0.641). A comparable result was observed for the adaptive exhaustion index (ρ = 0.072, *p* = 0.487, q = 0.652). Among individual checkpoint markers, SCORE2 was not significantly associated with CD4+ T-cell PD-1 expression (ρ = 0.049, *p* = 0.633, q = 0.760), CD4+ T-cell PD-L1 expression (ρ = −0.002, *p* = 0.988, q = 0.988), B-cell PD-1 expression (ρ = −0.005, *p* = 0.960, q = 0.974), or NK-cell PD-L1 expression (ρ = 0.020, *p* = 0.855, q = 0.938).

**Figure 6:**
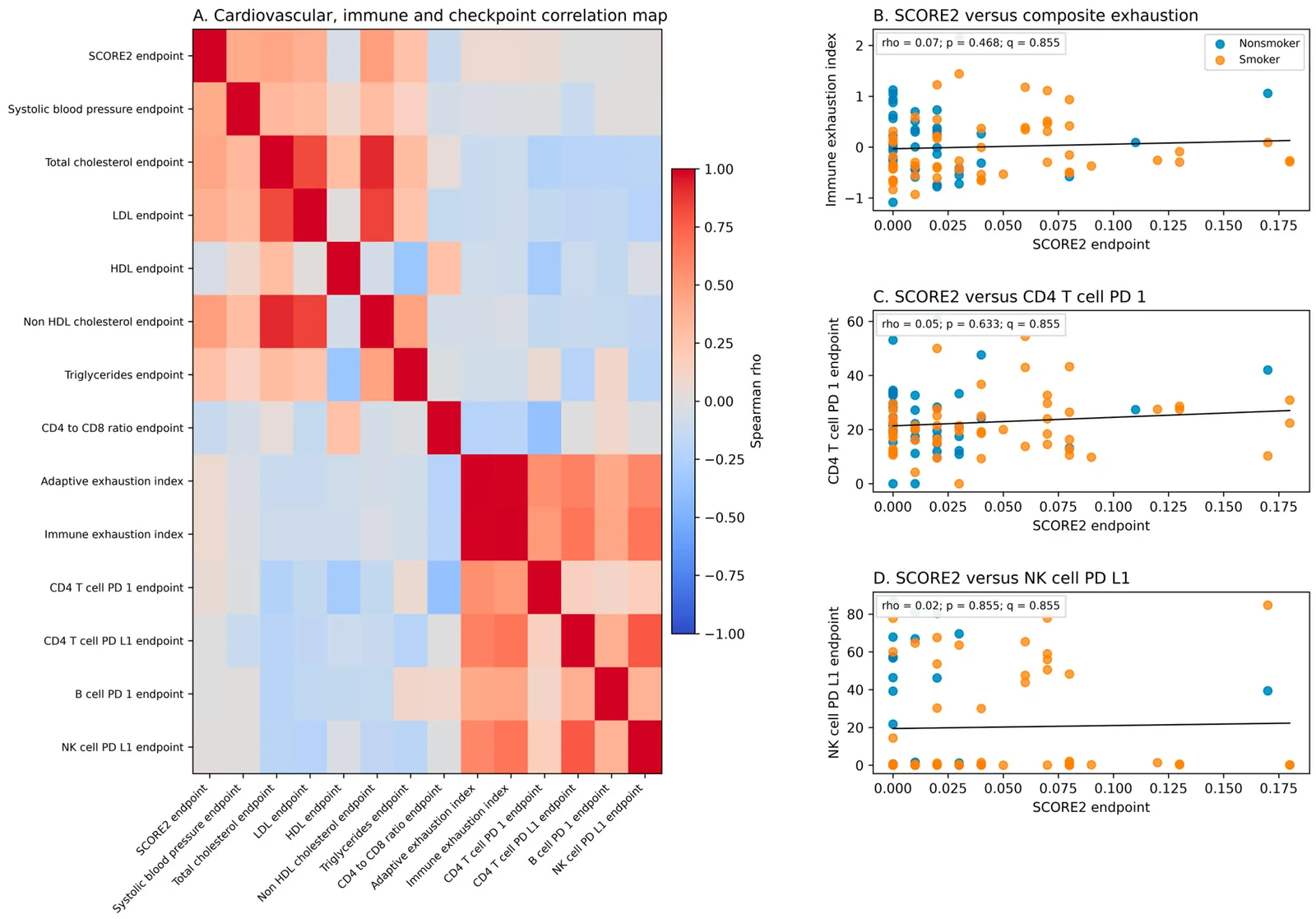
Correlation structure between SCORE2, cardiovascular parameters, immune-reconstitution indices, and endpoint PD-1/PD-L1 expression. (**A**) shows a Spearman rank correlation heatmap including endpoint SCORE2, systolic blood pressure, lipid parameters, CD4/CD8 ratio, composite exhaustion indices, and selected endpoint PD-1/PD-L1 markers. (**B**–**D**) show targeted scatterplots for endpoint SCORE2 versus the composite immune-exhaustion index, endpoint SCORE2 versus CD4+ T-cell PD-1 expression, and endpoint SCORE2 versus NK-cell PD-L1 expression. Spearman correlation coefficients, nominal *p*-values, and FDR-adjusted q-values are displayed in the corresponding panels. Points are colored by smoking status, and fitted lines are shown to aid visualization. In panel (**A**), red and blue indicate positive and negative Spearman correlations, respectively, with color intensity proportional to the absolute correlation coefficient; near-white cells indicate correlations close to zero. In panels (**B**–**D**), blue and orange points denote nonsmokers and smokers, respectively, and each point represents one participant. Black fitted lines are included for visualization only.

The heatmap also demonstrated internal coherence of the checkpoint-exhaustion measures. The adaptive and overall immune-exhaustion indices were strongly correlated with one another (ρ = 0.986, *p* < 0.001, q < 0.001), and the immune-exhaustion index correlated with several individual checkpoint markers, including CD4+ T-cell PD-L1 (ρ = 0.666), NK-cell PD-L1 (ρ = 0.669), CD4+ T-cell PD-1 (ρ = 0.498), and B-cell PD-1 (ρ = 0.441). The complete correlation structure and the targeted SCORE2–checkpoint relationships are presented in Figure 6.

### 3.5. Adjusted Models Confirmed Divergence Between Smoking and Immune-Exhaustion Markers

In models adjusted for prespecified clinical covariates, including age, sex, clinical group, ART category, HIV subtype category, and active HCV status, smoking was not significantly associated with the adaptive exhaustion index (β = −0.208; 95% confidence interval (CI): −0.494 to 0.077; *p* = 0.152; q = 0.413) or the overall immune-exhaustion index (β = −0.195; 95% CI: −0.482 to 0.092; *p* = 0.184; q = 0.413). The confidence intervals for both composite indices crossed zero, indicating no robust adjusted smoking-associated shift.

**Figure 7:**
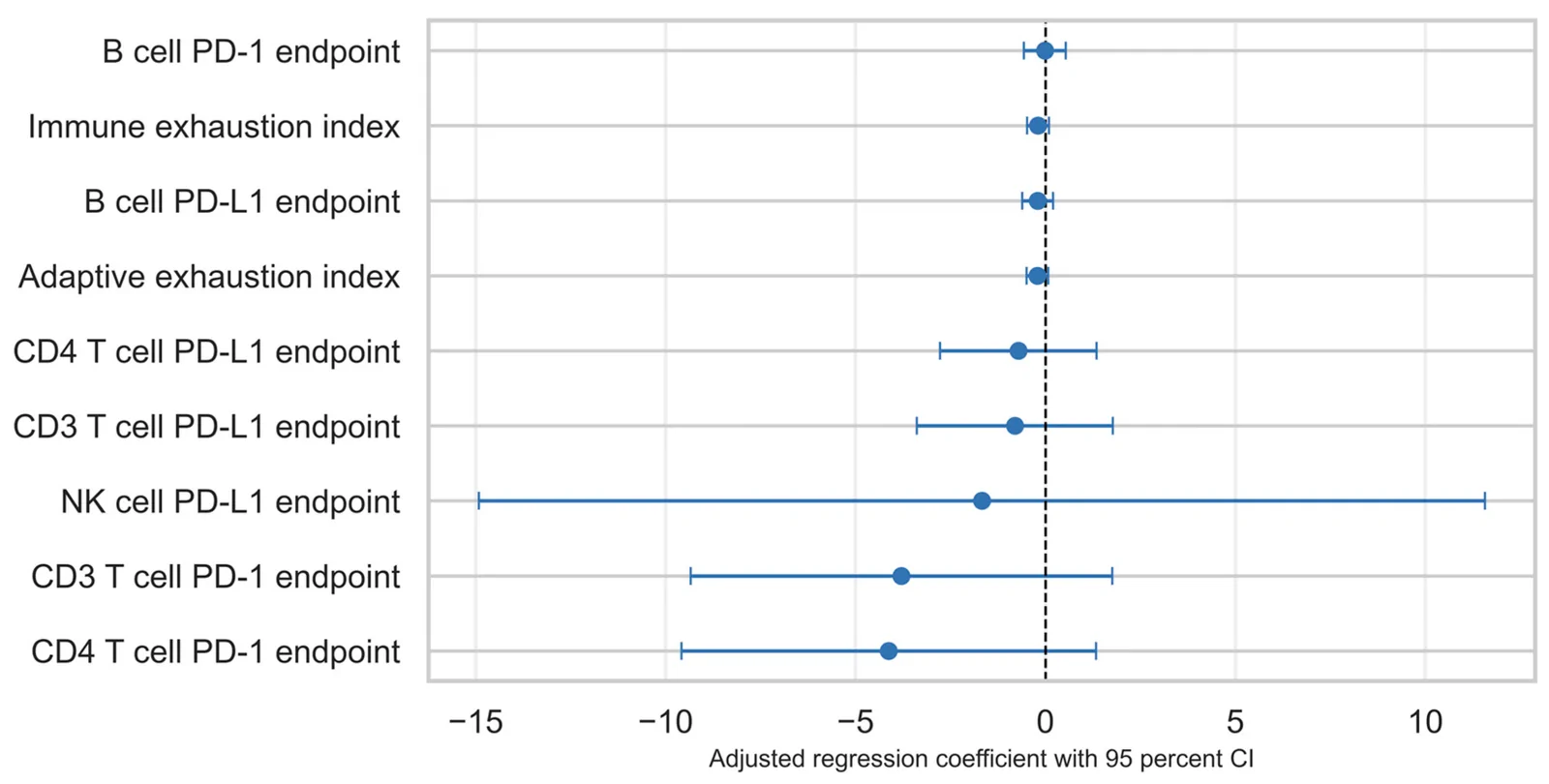
Adjusted regression estimates for the association between smoking status and endpoint checkpoint-defined immune-exhaustion outcomes. Forest plot showing adjusted ordinary least-squares regression coefficients for smoking status across composite and individual immune-exhaustion outcomes. Models were adjusted for covariates, including age, sex, clinical group, antiretroviral therapy category, HIV subtype category, and active HCV status. Points indicate adjusted regression coefficients and horizontal lines indicate 95% confidence intervals. The vertical dashed line indicates the null value (β = 0). Points represent adjusted regression coefficients, and horizontal lines represent 95% confidence intervals.

The same pattern was observed for individual checkpoint markers. Smoking was not significantly associated with CD3+ T-cell PD-1 expression (β = −3.79; 95% CI: −9.34 to 1.76; *p* = 0.181), CD4+ T-cell PD-1 expression (β = −4.12; 95% CI: −9.58 to 1.34; *p* = 0.139), CD3+ T-cell PD-L1 expression (β = −0.81; 95% CI: −3.39 to 1.78; *p* = 0.541), or CD4+ T-cell PD-L1 expression (β = −0.71; 95% CI: −2.77 to 1.35; *p* = 0.498). B-cell checkpoint markers also showed no significant adjusted association with smoking, including B-cell PD-1 (β = −0.02; 95% CI: −0.57 to 0.54; *p* = 0.957) and B-cell PD-L1 (β = −0.20; 95% CI: −0.61 to 0.20; *p* = 0.322). NK-cell PD-L1 showed a wide confidence interval, reflecting greater variability and lower complete-case availability, but no significant association was detected (β = −1.67; 95% CI: −14.92 to 11.57; *p* = 0.805). Across all modeled immune outcomes, FDR-adjusted q-values remained above 0.05, supporting the conclusion that smoking status did not independently explain endpoint checkpoint-expression differences. The adjusted smoking coefficients and their 95% confidence intervals are summarized in Figure 7. Complete estimates from the primary adjusted smoking models are provided in Supplementary Table S10.

### 3.6. Discordant Cardiovascular and Immune-Exhaustion Risk Profiles Were Identifiable at the Patient Level

Patient-level discordance analysis was performed using quartile-based thresholds for endpoint SCORE2 and the composite immune-exhaustion index. The upper quartile of SCORE2 was 4.25%, and the lower quartile was 0.0%. For the immune-exhaustion index, the upper-quartile threshold was 0.351 and the lower-quartile threshold was −0.426. Using these predefined thresholds, using these predefined thresholds, 72 of 96 participants (75.0%) were classified as intermediate, 14 of 96 (14.6%) had concordant extreme profiles, and 10 of 96 (10.4%) had discordant profiles. Thus, intermediate and concordant profiles together comprised 86 of 96 participants (89.6%). However, distinct discordant profiles were identifiable.

A high-cardiovascular-risk/high-exhaustion profile was observed in eight participants (8.3%), whereas a low-cardiovascular-risk/low-exhaustion profile was observed in six participants (6.3%). Importantly, discordant cardiovascular–immunological profiles were also present: four participants (4.2%) had high SCORE2 but low immune exhaustion, and six participants (6.3%) had low SCORE2 but high immune exhaustion. Together, these discordant groups represented 10 of 96 evaluable participants (10.4%), supporting the presence of clinically relevant mismatch between classical cardiovascular-risk classification and checkpoint-defined immune-exhaustion status.

**Figure 8:**
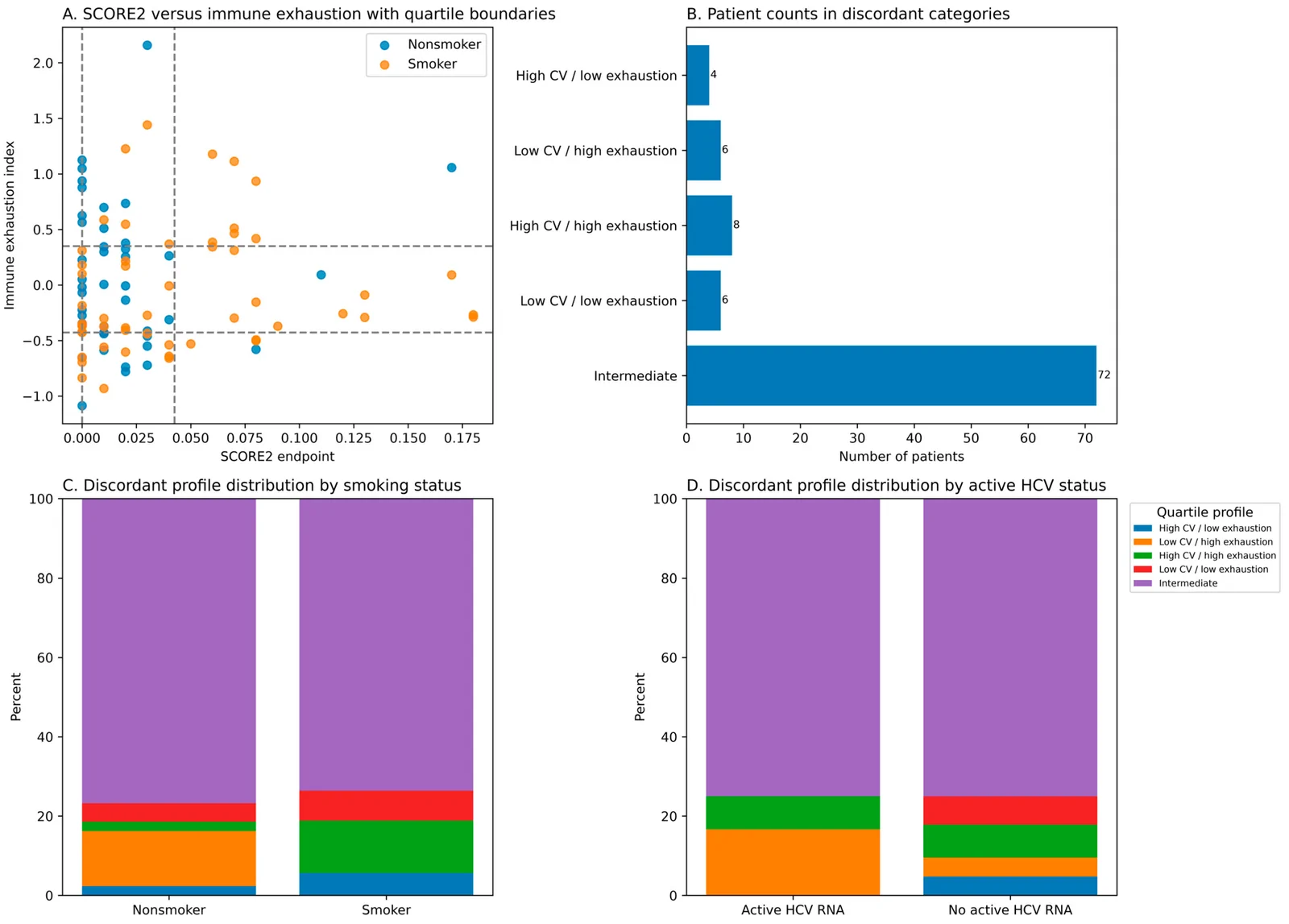
Patient-level discordance profiles based on endpoint SCORE2 and the composite immune-exhaustion index. (**A**) shows the relationship between endpoint SCORE2 and the composite immune-exhaustion index, with quartile-based thresholds used to define cardiovascular-risk and immune-exhaustion strata. (**B**) summarizes the number of participants across all quartile-defined profile categories, including the intermediate category. (**C**) displays the distribution of quartile-defined profile distribution by smoking status, and (**D**) displays the quartile-defined profile distribution by active HCV status. High cardiovascular risk and high immune exhaustion were defined using upper-quartile thresholds, whereas low cardiovascular risk and low immune exhaustion were defined using lower-quartile thresholds; remaining participants were classified as intermediate. In panel (**A**), dashed vertical lines denote the lower and upper quartiles of endpoint SCORE2 (0.0% and 4.25%, respectively), and dashed horizontal lines denote the lower and upper quartiles of the composite immune-exhaustion index (−0.426 and 0.351, respectively). Blue and orange points denote nonsmokers and smokers, respectively. Colors in panels (**C**,**D**) correspond to the quartile-defined profile categories shown in the embedded legend. CV, cardiovascular.

Stratification by smoking status showed that smokers were more frequently represented in high-cardiovascular-risk categories. Among smokers, 7 of 53 (13.2%) were classified as high cardiovascular risk/high exhaustion and 3 of 53 (5.7%) as high cardiovascular risk/low exhaustion. Among nonsmokers, 1 of 43 (2.3%) was classified as high cardiovascular risk/high exhaustion and 1 of 43 (2.3%) as high cardiovascular risk/low exhaustion. Conversely, the low-cardiovascular-risk/high-exhaustion profile was observed among nonsmokers only in this quartile-defined analysis (6 of 43, 14.0%). When stratified by active HCV status, 2 of 12 participants with active HCV RNA (16.7%) were classified as low cardiovascular risk/high exhaustion, while 1 of 12 (8.3%) showed high cardiovascular risk/high exhaustion. Among participants without active HCV RNA, discordant profiles were also observed, including high cardiovascular risk/low exhaustion in 4 of 84 participants (4.8%) and low cardiovascular risk/high exhaustion in 4 of 84 participants (4.8%). Individual-level and stratified quartile-defined cardiovascular–immune profiles are summarized in Figure 8.

### 3.7. Active HCV Status Did Not Explain the Primary Smoking–Exhaustion Dissociation—Sensitivity Analyses

After exclusion of HCV-RNA-positive participants, smoking was not significantly associated with the adaptive exhaustion index (β = −0.255; 95% CI: −0.558 to 0.047; *p* = 0.098; q = 0.395) or the overall immune-exhaustion index (β = −0.242; 95% CI: −0.545 to 0.060; *p* = 0.116; q = 0.395). These estimates were directionally comparable to the primary models that included active HCV status as a covariate, and the confidence intervals continued to cross zero.

**Figure 9:**
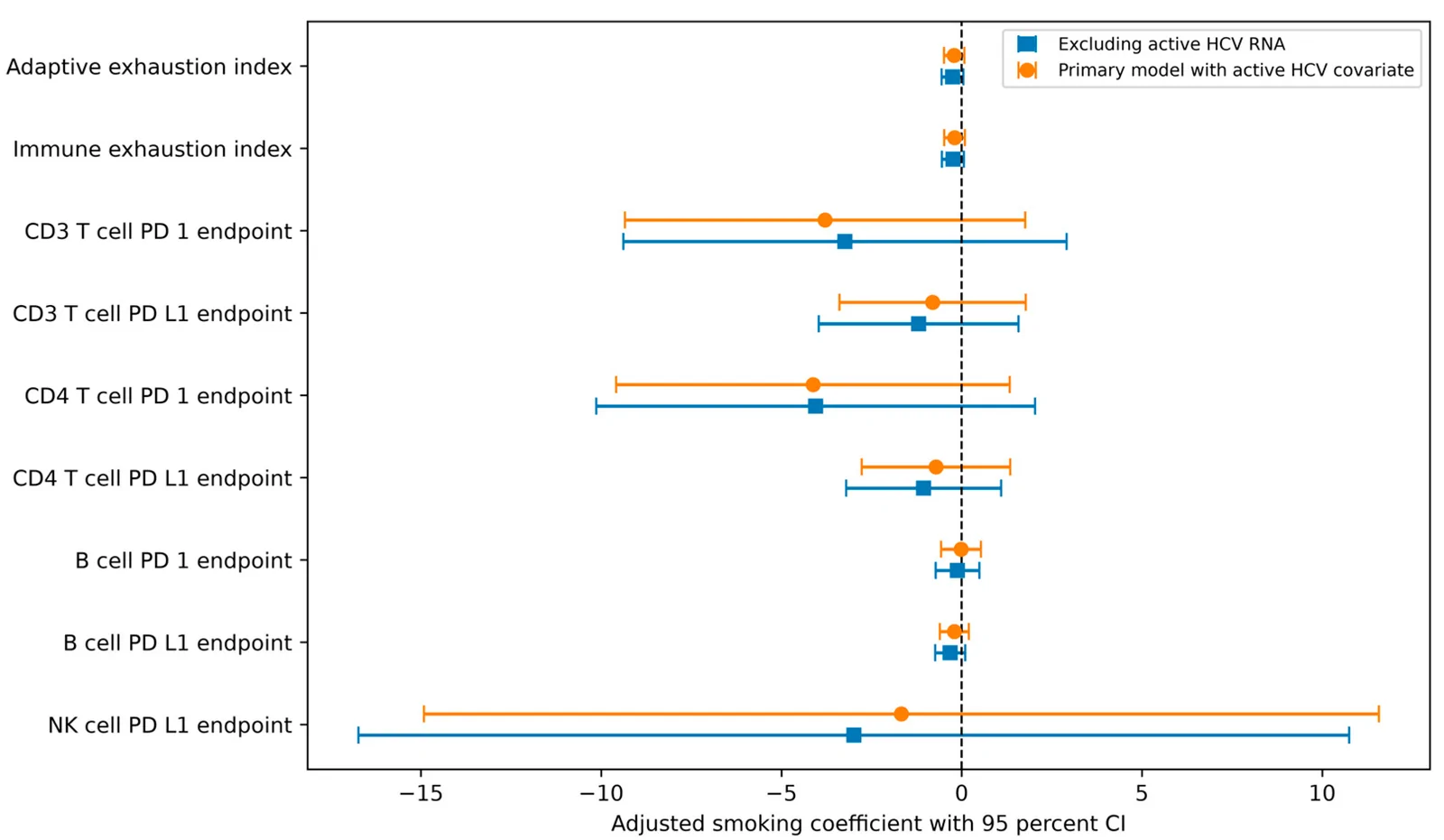
Sensitivity analysis of smoking-associated immune-exhaustion estimates after exclusion of participants with active HCV replication. Forest plot comparing adjusted smoking coefficients from the primary models, which included active HCV status as a covariate, with sensitivity models fitted after excluding HCV-RNA-positive participants. Outcomes include composite exhaustion indices and individual endpoint PD-1/PD-L1 markers. Points indicate adjusted coefficients and horizontal lines indicate 95% confidence intervals. Blue squares denote models fitted after exclusion of participants with active HCV RNA, whereas orange circles denote the primary models including active HCV status as a covariate. The vertical dashed line indicates the null value (β = 0); horizontal lines represent 95% confidence intervals.

The HCV-excluded sensitivity models similarly showed no significant association between smoking and individual endpoint checkpoint markers. For CD3+ T-cell PD-1, the smoking coefficient was −3.24 (95% CI: −9.38 to 2.91; *p* = 0.302), and for CD4+ T-cell PD-1 it was −4.05 (95% CI: −10.14 to 2.04; *p* = 0.192). PD-L1 markers also remained non-significant, including CD3+ T-cell PD-L1 (β = −1.19; 95% CI: −3.97 to 1.58; *p* = 0.398), CD4+ T-cell PD-L1 (β = −1.05; 95% CI: −3.20 to 1.10; *p* = 0.337), B-cell PD-L1 (β = −0.32; 95% CI: −0.73 to 0.10; *p* = 0.132), and NK-cell PD-L1 (β = −2.99; 95% CI: −16.73 to 10.75; *p* = 0.670). All FDR-adjusted q-values remained above 0.05. Therefore, exclusion of active HCV did not materially change the primary finding that smoking was not independently associated with higher checkpoint-defined immune exhaustion. The primary and HCV-excluded adjusted estimates are compared in Figure 9.

### 3.8. Exploratory Principal Component Analysis (PCA) Supported Partial Separation of Cardiovascular and Checkpoint-Exhaustion Variables

**Figure 10:**
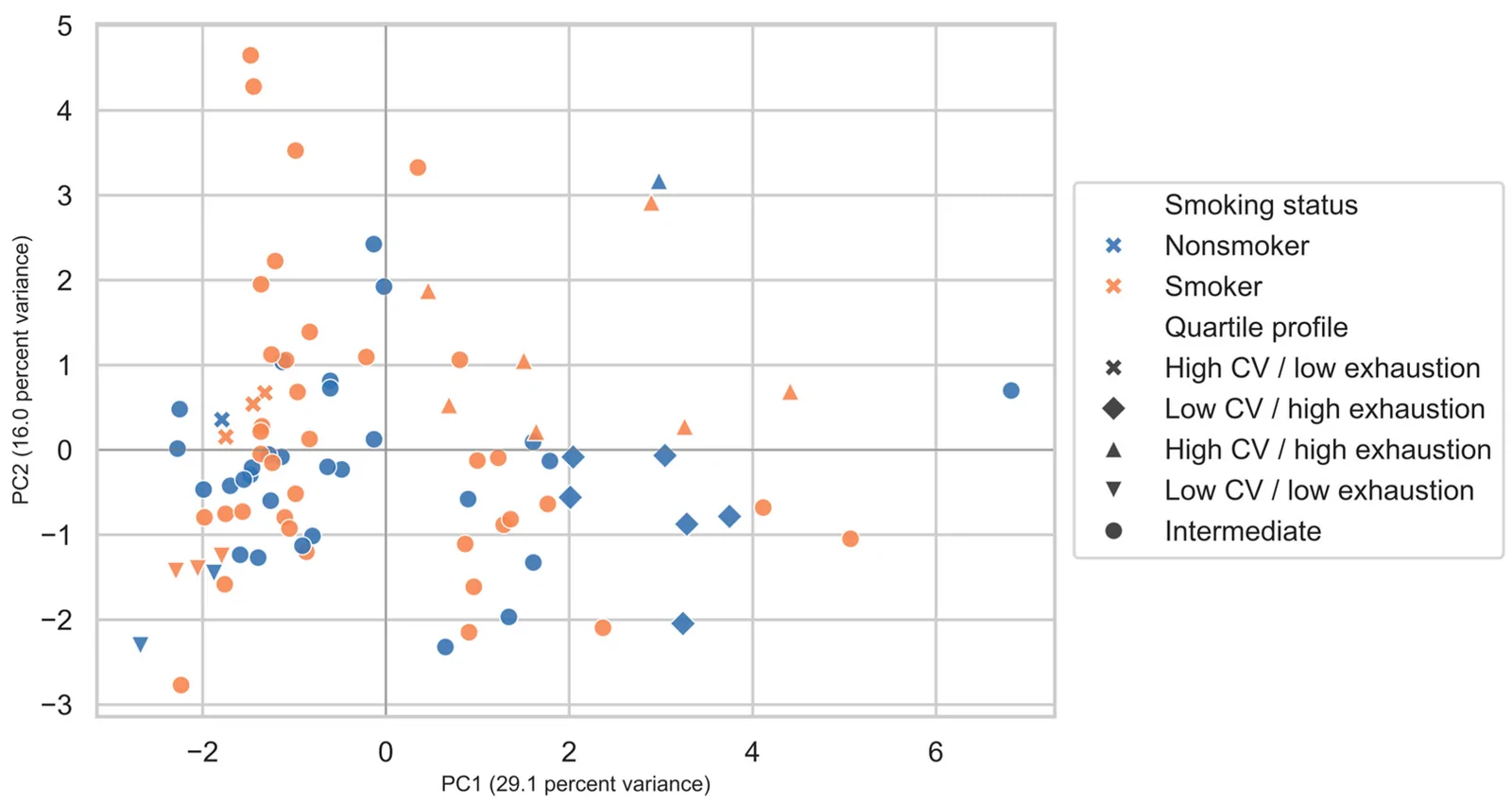
Principal component analysis of standardized cardiovascular, immune-reconstitution, and checkpoint-exhaustion variables. The scatterplot displays participants according to the first two principal components, with the percentage of explained variance shown on each axis. Point color denotes smoking status and point shape denotes quartile-defined discordance profile based on endpoint SCORE2 and the composite immune-exhaustion index. Blue and orange points denote nonsmokers and smokers, respectively. Point shapes denote the five quartile-defined cardiovascular–immune profile categories shown in the legend. Each point represents one complete-case participant. CV, cardiovascular; PC, principal component.

Exploratory principal component analysis was performed to visualize the multivariable structure of cardiovascular, immune-reconstitution, and checkpoint-exhaustion variables. PC1 ranged from −2.68 to 6.82 and PC2 from −2.77 to 4.65, indicating substantial interindividual heterogeneity in the combined cardiovascular–immune variable space. Smokers and nonsmokers showed broad overlap across the first two principal components. Median PC1 values were −0.97 in smokers and −0.61 in nonsmokers (Mann–Whitney U test, *p* = 0.775), and median PC2 values were 0.04 in smokers and −0.23 in nonsmokers (*p* = 0.213). Thus, smoking status alone did not generate a distinct PCA-defined cluster. In contrast, quartile-defined discordance profiles showed greater spatial organization across the PCA plane. Profiles characterized by high immune exhaustion tended to occupy more positive PC1 values, including high cardiovascular risk/high exhaustion (median PC1 2.27) and low cardiovascular risk/high exhaustion (median PC1 3.14). Profiles characterized by low immune exhaustion were positioned toward more negative PC1 values, including high cardiovascular risk/low exhaustion (median PC1 −1.60) and low cardiovascular risk/low exhaustion (median PC1 −2.05). Exploratory group comparisons across discordance profiles showed significant differences in both PC1 and PC2 coordinates (Kruskal–Wallis *p* < 0.001 for both axes). The distribution of participants across the first two principal components is shown in Figure 10.

### 3.9. Sensitivity Analyses Addressing Extreme Values, Missingness, and SCORE2 Age Range

Observations beyond the 1.5 × IQR boxplot whiskers were identified for the cardiovascular and immune-marker displays. These values were reviewed for plausibility and no biologically impossible flow-cytometric percentages or implausible cardiovascular/lipid values were identified. Therefore, no observations were removed from the primary analyses solely because they appeared beyond boxplot whiskers. Variable-specific whisker thresholds and the numbers of observations beyond the whiskers are provided in Supplementary Table S4.

Winsorization of endpoint checkpoint markers at the fifth and 95th percentiles did not materially change the primary findings. Endpoint SCORE2 remained weakly and non-significantly correlated with the winsorized T/B-cell immune-exhaustion index (Spearman ρ = 0.052, *p* = 0.614, q = 0.705). In the adjusted smoking model, smoking was not significantly associated with the winsorized T/B-cell exhaustion index (β = −0.255; 95% CI: −0.624 to 0.113; *p* = 0.174). Quartile-defined discordance based on the winsorized index identified 11 discordant participants, compared with 10 in the primary quartile-defined analysis. The results of the complete-case, HCV-excluded, subgroup, alternative ART-classification, and winsorized sensitivity analyses are summarized in Supplementary Table S5.

Regression influence diagnostics did not indicate that the principal conclusion was driven by a small number of high-influence observations. When adjusted models were repeated after excluding observations with Cook’s distance greater than 4/n, smoking remained non-significantly associated with the composite immune-exhaustion indices and did not show a consistent positive association with individual endpoint checkpoint markers after false-discovery-rate correction. Detailed influence diagnostics and estimates obtained after exclusion of potentially influential observations are presented in Supplementary Table S6.

The full cohort included 100 participants, but complete-case numbers varied by analysis. The primary T/B-cell checkpoint composite index and discordance analysis included 96 participants, whereas NK-cell analyses included 89 participants. The age distribution was also evaluated because SCORE2 is conventionally intended for individuals aged 40–69 years. Median age was 41.5 years (IQR: 34.8–47.0; range 23–73); 45 participants were younger than 40 years, 54 were aged 40–69 years, and one was older than 69 years. In the age-restricted subgroup aged 40–69 years, endpoint SCORE2 remained uncorrelated with the T/B-cell composite exhaustion index (Spearman ρ = −0.045, *p* = 0.753). The cohort age distribution and SCORE2 age-range eligibility are summarized in Supplementary Table S7. Detailed age-restricted sensitivity analyses are presented in Supplementary Table S8, whereas the distribution across 10-year age bands is shown in Supplementary Table S9 and Supplementary Figure S1.

## 4. Discussion

In this study, we evaluated whether conventional cardiovascular risk, expressed by SCORE2, corresponds to checkpoint-defined immune exhaustion in people with HIV. The main finding is that these two dimensions of residual risk were only weakly aligned. Smoking was associated with a clear increase in SCORE2-defined cardiovascular risk, but this was not accompanied by a consistent increase in PD-1/PD-L1 expression on T-cell, B-cell, or NK-cell compartments. Moreover, endpoint SCORE2 correlated with expected cardiovascular parameters, including systolic blood pressure and lipid variables, but showed low or absent correlations with composite and individual immune-exhaustion markers. These findings indicate limited contemporaneous alignment between SCORE2 and the measured PD-1/PD-L1 checkpoint-expression profiles; they do not establish biological independence between these domains.

The increased burden of cardiovascular disease in PWH is well established. Previous cohort studies demonstrated higher rates of myocardial infarction and cardiovascular disease among HIV-positive individuals compared with HIV-negative populations, even after adjustment for traditional cardiovascular risk factors [27,28,29]. A systematic review and meta-analysis further confirmed that the global burden of HIV-associated cardiovascular disease has increased substantially over time [29]. In this context, cardiovascular prevention has become a central component of long-term HIV care.

The observed association between smoking and higher SCORE2 was expected and clinically relevant. Smoking is one of the strongest modifiable cardiovascular risk factors in PWH and is directly incorporated into SCORE2. Previous studies showed that smoking contributes substantially to excess mortality in HIV-positive populations, while smoking cessation is associated with a reduction in cardiovascular event rates [13,14]. In our cohort, smokers had higher baseline and endpoint SCORE2 values than nonsmokers, whereas individual lipid and blood pressure parameters did not show a uniform smoking-related shift. This indicates that smoking status itself was a major driver of calculated cardiovascular risk, rather than a broad separation across all measured classical cardiovascular variables. However, smoking was not associated with higher checkpoint-defined immune exhaustion. Neither the composite immune-exhaustion indices nor individual PD-1/PD-L1 markers differed significantly between smokers and nonsmokers after correction for multiple testing. This argues against a simple model in which a major behavioral cardiovascular risk factor automatically translates into a parallel increase in checkpoint-defined immune dysfunction. Smoking may promote cardiovascular disease through mechanisms such as endothelial dysfunction, oxidative stress, thrombosis, inflammation, and lipid oxidation [6,14], but these mechanisms may not be directly reflected by PD-1/PD-L1 expression on circulating lymphocyte subsets. Therefore, checkpoint-defined exhaustion should not be treated as a surrogate of classical cardiovascular risk.

The dissociation between SCORE2 and immune-exhaustion markers is consistent with broader concerns regarding cardiovascular risk prediction in PWH. SCORE2 was developed to estimate 10-year fatal and non-fatal cardiovascular risk in the European general population [6]. HIV-specific tools, including the D:A:D risk equation, incorporate selected HIV-related and treatment-related variables, but their clinical performance remains imperfect [7]. A systematic review and meta-analysis showed that both general-population and HIV-specific cardiovascular risk models have only moderate discrimination in PWH and may underpredict cardiovascular risk [8,30]. The REPRIEVE trial further demonstrated that pitavastatin reduced major adverse cardiovascular events in PWH with low-to-moderate predicted cardiovascular risk, suggesting that conventional risk thresholds may not fully capture cardiovascular vulnerability in treated HIV infection [9]. Our data extend this concept by showing that SCORE2 may also fail to reflect a distinct checkpoint-defined immune phenotype. Persistent immune activation, inflammation, and coagulation abnormalities may partly explain why cardiovascular risk in PWH is not fully captured by classical algorithms. Inflammatory and coagulation biomarkers, including IL-6 and D-dimer, have been associated with mortality, serious non-AIDS events, and cardiovascular complications in HIV-positive individuals, including patients receiving suppressive ART [15,31]. The CD4+/CD8+ ratio has also been proposed as a marker of immune senescence and residual immune dysfunction, and low values have been associated with serious non-AIDS events [16,17,18]. These observations support the concept that cardiovascular vulnerability in PWH may arise from both classical and immune-mediated pathways.

PD-1/PD-L1 expression has well-established relevance in HIV-related immune dysfunction. PD-1 is upregulated on HIV-specific T cells, correlates with impaired T-cell function, viral replication, and disease progression, and may remain elevated despite antiretroviral therapy in selected patients [19,20,21]. Checkpoint-expressing CD4+ T cells are also enriched for persistent HIV during suppressive ART, linking immune checkpoint biology with viral persistence and chronic immune dysregulation [22]. Previous studies from our group have similarly suggested that PD-1/PD-L1 expression may persist despite virological suppression and immune restoration, and that immune recovery defined by CD4+ T-cell count or CD4+/CD8+ ratio may not fully normalize checkpoint-defined immune phenotypes [24,25,26]. In this context, the current findings support the interpretation that PD-1/PD-L1 expression captures an immunological axis that is not reducible to SCORE2-defined cardiovascular risk.

One of the most clinically relevant observations was the identification of discordant cardiovascular–immune profiles. A subset of participants had high SCORE2 but low immune exhaustion, whereas another subset had low SCORE2 but high immune exhaustion. These discordant groups represented a minority of the cohort, but their presence is conceptually important. Patients with high cardiovascular risk and low checkpoint-defined exhaustion may primarily require aggressive management of classical risk factors. Conversely, patients with low cardiovascular risk but high checkpoint-defined exhaustion may represent a group in whom conventional cardiovascular algorithms underestimate broader immune dysregulation. At present, these profiles should be interpreted as exploratory biological phenotypes rather than validated clinical risk categories. However, they provide a rationale for future studies integrating cardiovascular, metabolic, inflammatory, and immunophenotypic markers in PWH.

Active HCV infection did not explain the primary dissociation between smoking and immune exhaustion. After exclusion of HCV-RNA-positive participants, smoking remained unassociated with composite and individual checkpoint outcomes. This strengthens the robustness of the main observation. Nevertheless, the small number of participants with active HCV limits the ability to draw firm conclusions regarding the independent effect of HCV replication on cardiovascular–immune discordance.

## 5. Limitations of the Study

This study has limitations. It was a single-center observational study with a moderate sample size, which limits statistical power, particularly in subgroup and sensitivity analyses. The cohort included both newly diagnosed and long-term-treated PWH, introducing clinical heterogeneity. SCORE2 is a conventional cardiovascular risk tool and does not incorporate HIV-specific factors such as duration of infection, nadir CD4+ T-cell count, cumulative viremia, immune activation, inflammatory biomarkers, coinfections, or detailed cumulative ART exposure. The study did not include prospective cardiovascular endpoints; therefore, the identified discordance profiles cannot be interpreted as predictors of clinical cardiovascular events. Checkpoint-defined exhaustion was assessed phenotypically by flow cytometry, without functional T-cell assays or direct inflammatory biomarker profiling. Finally, the composite exhaustion index was exploratory and requires external validation.

## 6. Conclusions

In conclusion, SCORE2 and PD-1/PD-L1 checkpoint-expression profiles showed limited contemporaneous alignment in this secondary cohort analysis, without establishing biological independence or a validated new category of clinical risk. Smoking strongly influenced calculated cardiovascular risk, but did not translate into a parallel increase in checkpoint-defined immune exhaustion. The presence of discordant cardiovascular–immune profiles suggests that conventional risk algorithms may not fully capture immune dysregulation in treated HIV infection. Future prospective studies should determine whether combined cardiovascular and immunophenotypic profiling improves prediction of non-AIDS comorbidities and identifies PWH who may benefit from more individualized monitoring and prevention strategies.

## Data Availability

De-identified data supporting the present analyses are available from the corresponding author upon reasonable request, subject to institutional and ethical requirements. For data requests, please contact the corresponding author: karolina.skonieczna.zydecka@pum.edu.pl.

## Supplementary Materials

The following supporting information can be downloaded at https://www.mdpi.com/article/10.3390/pathogens15080871/s1, Table S1: Demographic, clinical, cardiovascular, and immunological characteristics of the cohort; Table S2: Missingness by variable; Table S3: Participant numbers included in the main analyses; Table S4: Observations beyond boxplot whiskers; Table S5: Robustness of the primary findings across sensitivity analyses; Table S6: Regression influence diagnostics and Cook’s-distance sensitivity analysis; Table S7: Age distribution and SCORE2 age-range eligibility; Table S8: Sensitivity analyses restricted to participants aged 40–69 years; Table S9: Age distribution of the cohort; Table S10: Primary adjusted smoking models; Figure S1: Age distribution of the cohort.
