# Supplementary material for: Patient-Level Discordance Between SCORE2 Cardiovascular Risk and PD-1/PD-L1 Checkpoint-Expression Profiles in People Living with HIV: A Secondary Cohort Analysis
Source: Pathogens. 2026 Aug 20;15(8):871. doi: 10.3390/pathogens15080871 (PMC13516168; doi:10.3390/pathogens15080871)
Supplement: Supplementary file 1 [file pathogens-15-00871-s001.zip › pathogens-4480914-supplementary.pdf]

Table S1. Demographic, clinical, cardiovascular, and immunological characteristics of the cohort.

Characteristic	Overall	Smokers	Nonsmokers	N available
Age, years	41.5 (34.8-47.0)	42.0 (37.0-46.8)	39.5 (33.0-46.8)	100
Sex: Male	56/100 (56.0%)	38/54 (70.4%)	18/46 (39.1%)	100
Sex: Female	44/100 (44.0%)	16/54 (29.6%)	28/46 (60.9%)	100
Smoking status: Smoker	54/100 (54.0%)	54/54 (100.0%)	0/46 (0.0%)	100
Smoking status: Nonsmoker	46/100 (46.0%)	0/54 (0.0%)	46/46 (100.0%)	100
Clinical group: Newly diagnosed	58/100 (58.0%)	31/54 (57.4%)	27/46 (58.7%)	100
Clinical group: Long-term treated	42/100 (42.0%)	23/54 (42.6%)	19/46 (41.3%)	100
ART category: INSTI+TAF	51/99 (51.5%)	27/54 (50.0%)	24/46 (52.2%)	99
ART category: INSTI+TDF	37/99 (37.4%)	22/54 (40.7%)	15/46 (32.6%)	99
ART category: INSTI	5/99 (5.1%)	3/54 (5.6%)	2/46 (4.3%)	99
ART category: TAF	3/99 (3.0%)	1/54 (1.9%)	2/46 (4.3%)	99
ART category: non INSTI non TAF	3/99 (3.0%)	1/54 (1.9%)	2/46 (4.3%)	99
HIV subtype category: A6	44/100 (44.0%)	27/54 (50.0%)	17/46 (37.0%)	100
HIV subtype category: B	22/100 (22.0%)	16/54 (29.6%)	6/46 (13.0%)	100
HIV subtype category: Other	4/100 (4.0%)	0/54 (0.0%)	4/46 (8.7%)	100
HIV subtype category: Unknown	30/100 (30.0%)	11/54 (20.4%)	19/46 (41.3%)	100
Active HCV status: Active HCV RNA	12/100 (12.0%)	8/54 (14.8%)	4/46 (8.7%)	100
Active HCV status: No active HCV RNA	88/100 (88.0%)	46/54 (85.2%)	42/46 (91.3%)	100
CD4+ endpoint, cells/µL	603.5 (415.2-834.5)	610.5 (355.2-843.5)	596.5 (439.2-765.2)	100
CD8+ endpoint, cells/µL	741.5 (531.2-990.5)	737.0 (517.0-963.0)	755.0 (532.0-991.0)	98
CD4+/CD8+ endpoint	0.86 (0.48-1.25)	0.88 (0.49-1.32)	0.85 (0.48-1.21)	98
SCORE2 endpoint, %	2.0 (0.0-4.0)	3.5 (1.0-7.0)	1.0 (0.0-2.0)	100
Systolic BP endpoint, mmHg	131.5 (125.0-147.0)	131.0 (125.2-142.0)	133.5 (125.2-149.0)	100
LDL endpoint, mg/dL	123.0 (93.0-139.0)	118.5 (96.8-136.0)	125.0 (86.8-139.8)	100
HDL endpoint, mg/dL	50.3 (42.3-59.4)	50.0 (42.1-57.3)	51.2 (46.2-62.5)	100
Triglycerides endpoint, mg/dL	98.0 (75.0-152.2)	108.5 (77.0-176.0)	90.0 (74.2-130.0)	100
Total cholesterol endpoint, mg/dL	175.0 (150.0-202.0)	175.0 (152.5-195.8)	174.0 (148.2-202.0)	100
Non-HDL cholesterol endpoint, mg/dL	125.2 (99.4-145.6)	125.2 (100.0-154.2)	121.9 (94.1-141.9)	100
T/B-cell exhaustion index	-0.170 (-0.394-0.303)	-0.221 (-0.421-0.188)	0.051 (-0.379-0.458)	96
Expanded T/B/NK exhaustion index	-0.184 (-0.426-0.369)	-0.262 (-0.417-0.335)	-0.067 (-0.432-0.445)	89

Values are median (IQR) or n/N (%).

Supplementary Table S2. Missingness by variable.

Variable	Non-missing n	Missing n	Missing (%)
Age	100	0	0.0%
Sex	100	0	0.0%
Smoking status	100	0	0.0%
Clinical group	100	0	0.0%
ART category	99	1	1.0%
HIV subtype	70	30	30.0%
Active HCV RNA	30	70	70.0%
CD4+ endpoint	100	0	0.0%
CD8+ endpoint	98	2	2.0%
CD4+/CD8+ endpoint	98	2	2.0%
SCORE2 endpoint	100	0	0.0%
Systolic BP endpoint	100	0	0.0%
LDL endpoint	100	0	0.0%
HDL endpoint	100	0	0.0%
Triglycerides endpoint	100	0	0.0%
Total cholesterol endpoint	100	0	0.0%
Non-HDL cholesterol endpoint	100	0	0.0%
T/B exhaustion index	96	4	4.0%
Expanded T/B/NK exhaustion index	89	11	11.0%
NK-cell PD-L1 endpoint	89	11	11.0%
NK-cell frequency endpoint	89	11	11.0%

Missingness is calculated relative to the full cohort (N=100).

Supplementary Table S3. Participant numbers included in main analyses.

Analysis/Model	Required variables	N included
Full cohort description	Demographic/clinical variables	100
cardiovascular smoking comparisons	Smoking status plus SCORE2/lipids/BP	100
immune-marker smoking comparisons	Smoking status plus T/B-cell checkpoint markers	96
NK-cell analyses	NK-cell frequency and/or NK-cell PD-L1	89
Primary discordance analysis	Endpoint SCORE2 plus T/B-cell exhaustion index	96
Primary adjusted smoking models (T/B outcomes)	Outcome, smoking, age, sex, clinical group, ART, HIV subtype, active HCV	96
Primary adjusted smoking model (NK-cell PD-L1)	NK-cell PD-L1 plus covariates	89
HCV-excluded adjusted models (T/B outcomes)	No active HCV plus outcome/covariates	84
HCV-excluded NK-cell PD-L1 model	No active HCV plus NK-cell PD-L1/covariates	77
PCA displayed analysis	Complete cardiovascular/immune variables for displayed PCA	89
SCORE2 age-eligible sensitivity	Age 40-69 plus SCORE2 and immune index	54

N varies according to complete-case availability for each analysis.

Supplementary Table S4. Observations beyond boxplot whiskers.

Figure	Variable	Smoking group	N available	Q1	Q3	Lower whisker threshold	Upper whisker threshold	N below lower whisker	N above upper whisker	Total beyond whiskers	Observed min	Observed max
Figure 4	SCORE2 endpoint	Nonsmoker	46	0,000	0,020	-0,030	0,050	0	3	3	0,000	0,170
Figure 4	SCORE2 endpoint	Smoker	54	0,010	0,070	-0,080	0,160	0	3	3	0,000	0,180
Figure 4	SCORE2 baseline	Nonsmoker	46	0,000	0,020	-0,030	0,050	0	3	3	0,000	0,210
Figure 4	SCORE2 baseline	Smoker	54	0,010	0,070	-0,080	0,160	0	1	1	0,000	0,190
Figure 4	Systolic BP endpoint	Nonsmoker	46	125,250	149,000	89,625	184,625	0	0	0	116,000	177,000
Figure 4	Systolic BP endpoint	Smoker	54	125,250	142,000	100,125	167,125	0	4	4	110,000	199,000
Figure 4	LDL endpoint	Nonsmoker	46	86,750	139,750	7,250	219,250	0	0	0	59,000	189,000
Figure 4	LDL endpoint	Smoker	54	96,750	136,000	37,875	194,875	0	3	3	45,000	235,000
Figure 4	HDL endpoint	Nonsmoker	46	46,250	62,450	21,950	86,750	0	0	0	25,700	81,300
Figure 4	HDL endpoint	Smoker	54	42,075	57,300	19,238	80,138	1	1	2	9,700	84,400
Figure 4	Triglycerides endpoint	Nonsmoker	46	74,250	130,000	-9,375	213,625	0	2	2	39,000	468,000
Figure 4	Triglycerides endpoint	Smoker	54	77,000	176,000	-71,500	324,500	0	5	5	38,000	512,000
Figure 5	T/B-cell exhaustion index	Nonsmoker	43	-0,379	0,458	-1,633	1,712	0	1	1	-1,151	2,231
Figure 5	T/B-cell exhaustion index	Smoker	53	-0,421	0,188	-1,335	1,102	0	3	3	-0,971	1,429
Figure 5	CD3+ T-cell PD-1 endpoint (%)	Nonsmoker	43	20,100	35,300	-2,700	58,100	0	0	0	0,000	50,300
Figure 5	CD3+ T-cell PD-1 endpoint (%)	Smoker	53	19,000	28,000	5,500	41,500	0	2	2	7,800	69,600
Figure 5	CD4+ T-cell PD-1 endpoint (%)	Nonsmoker	43	16,650	29,600	-2,775	49,025	0	2	2	0,000	61,000
Figure 5	CD4+ T-cell PD-1 endpoint (%)	Smoker	53	13,800	27,200	-6,300	47,300	0	2	2	0,000	54,500
Figure 5	B-cell PD-1 endpoint (%)	Nonsmoker	43	0,250	1,150	-1,100	2,500	0	4	4	0,000	5,500
Figure 5	B-cell PD-1 endpoint (%)	Smoker	53	0,300	0,900	-0,600	1,800	0	6	6	0,000	12,700
Figure 5	CD4+ T-cell PD-L1 endpoint (%)	Nonsmoker	43	0,100	7,550	-11,075	18,725	0	0	0	0,000	16,100
Figure 5	CD4+ T-cell PD-L1 endpoint (%)	Smoker	53	0,000	5,400	-8,100	13,500	0	2	2	0,000	15,200
Figure 5	NK-cell PD-L1 endpoint (%)	Nonsmoker	39	0,150	42,800	-63,825	106,775	0	0	0	0,000	87,300
Figure 5	NK-cell PD-L1 endpoint (%)	Smoker	50	0,200	48,125	-71,688	120,013	0	0	0	0,000	84,700

Supplementary Table S5. Robustness of primary findings across sensitivity analyses.

Sensitivity analysis	Index used	Threshold type	N with SCORE2 and index	SCORE2 versus index Spearman rho	SCORE2 versus index p-value	Smoking beta	Smoking lower 95 CI	Smoking upper 95 CI	Smoking p-value	Smoking model N	SCORE2 beta	SCORE2 lower 95 CI	SCORE2 upper 95 CI	SCORE2 p-value	SCORE2 model N	High CV low exhaustion count	Low CV high exhaustion count	Any discordance count	Non classifiable count	SCORE2 versus index FDR q-value	Smoking model FDR q-value	SCORE2 model FDR q-value
Expanded T/B/NK exhaustion index	Immune exhaustion index	quartile	96	0,075	0,468	-0,205	-0,538	0,127	0,226	96	1,179	-1,565	3,924	0,400	96	4	6	10	4	0,696	0,308	0,617
T/B-cell exhaustion index only	Adaptive exhaustion index	quartile	96	0,072	0,487	-0,222	-0,547	0,104	0,182	96	1,257	-1,572	4,086	0,384	96	4	7	11	4	0,696	0,308	0,617
Quartile-defined discordance	Immune exhaustion index	quartile	96	0,075	0,468	-0,205	-0,538	0,127	0,226	96	1,179	-1,565	3,924	0,400	96	4	6	10	4	0,696	0,308	0,617
Median-defined discordance	Immune exhaustion index	median	96	0,075	0,468	-0,205	-0,538	0,127	0,226	96	1,179	-1,565	3,924	0,400	96	25	19	44	4	0,696	0,308	0,617
Complete-case analysis	Immune exhaustion index	quartile	89	0,051	0,634	-0,173	-0,524	0,177	0,333	89	0,738	-2,112	3,588	0,612	89	4	6	10	0	0,705	0,370	0,680
Exclusion of active HCV	Immune exhaustion index	quartile	84	0,115	0,297	-0,234	-0,604	0,136	0,215	84	1,557	-1,519	4,633	0,321	84	4	4	8	4	0,696	0,308	0,617
Newly diagnosed only	Immune exhaustion index	quartile	56	0,017	0,901	-0,311	-0,836	0,214	0,246	56	-0,744	-4,368	2,881	0,688	56	3	4	7	2	0,901	0,308	0,688
Long-term treated only	Immune exhaustion index	quartile	40	0,195	0,228	0,013	-0,571	0,597	0,965	40	2,394	-3,577	8,365	0,432	40	1	4	5	2	0,696	0,965	0,617
Alternative ART grouping INSTI-based versus non-INSTI	Immune exhaustion index	quartile	96	0,075	0,468	-0,207	-0,549	0,134	0,234	96	1,392	-1,456	4,239	0,338	96	4	6	10	4	0,696	0,308	0,617
Winsorized immune markers	Winsorized immune exhaustion index	quartile	96	0,052	0,614	-0,255	-0,624	0,113	0,174	96	1,053	-2,358	4,464	0,545	96	4	7	11	4	0,705	0,308	0,680

Supplementary Table S6. Regression influence diagnostics and Cook-distance sensitivity analysis.

Outcome	Primary model N	Maximum Cook distance	N with Cook distance > 4/n	N high leverage > 2p/n	Primary smoking beta	Primary 95% CI low	Primary 95% CI high	Primary p	Primary q	Cook-excluded N	Cook-excluded smoking beta	Cook-excluded 95% CI low	Cook-excluded 95% CI high	Cook-excluded p	Cook-excluded q	Cook-excluded note
T/B-cell exhaustion index	96	0,140	7	13	-0,225	-0,565	0,115	0,195	0,565	89	-0,160	-0,447	0,127	0,276	0,827	excluded Cook > 4/n (n excluded=7)
Expanded T/B/NK exhaustion index	89	0,207	5	11	-0,191	-0,562	0,180	0,314	0,565	84	-0,116	-0,421	0,190	0,458	0,975	excluded Cook > 4/n (n excluded=5)
CD3+ T-cell PD-1 endpoint (%)	96	0,175	7	13	-4,063	-9,269	1,142	0,126	0,565	89	-6,176			1,000	1,000	excluded Cook > 4/n (n excluded=7)
CD3+ T-cell PD-L1 endpoint (%)	96	0,259	2	13	-0,362	-3,102	2,379	0,796	0,855	94	0,114	-2,410	2,638	0,929	1,000	excluded Cook > 4/n (n excluded=2)
CD4+ T-cell PD-1 endpoint (%)	96	0,078	5	13	-5,111	-10,643	0,421	0,070	0,565	91	-4,639	-9,030	-0,249	0,038	0,345	excluded Cook > 4/n (n excluded=5)
CD4+ T-cell PD-L1 endpoint (%)	96	0,434	10	13	-0,395	-2,830	2,041	0,751	0,855	86	-0,517	-2,620	1,586	0,630	0,975	excluded Cook > 4/n (n excluded=10)
B-cell PD-1 endpoint (%)	96	0,340	3	13	-0,075	-0,885	0,734	0,855	0,855	93	-0,093	-0,494	0,308	0,650	0,975	excluded Cook > 4/n (n excluded=3)
B-cell PD-L1 endpoint (%)	96	0,181	5	13	-0,283	-0,798	0,232	0,282	0,565	91	-0,169	-0,417	0,078	0,180	0,810	excluded Cook > 4/n (n excluded=5)
NK-cell PD-L1 endpoint (%)	89	0,190	8	11	-2,077	-17,662	13,508	0,794	0,855	81	0,813	-13,167	14,793	0,909	1,000	excluded Cook > 4/n (n excluded=8)

High leverage was defined as >2p/n. Potential influence was defined as Cook distance >4/n.

Supplementary Table S7. Age distribution and SCORE2 age-range eligibility.

Measure	Value
Median age (IQR), years	41.5 (34.8-47.0)
Minimum age, years	23
Maximum age, years	73
Participants younger than 40 years	45/100 (45.0%)
Participants aged 40-69 years	54/100 (54.0%)
Participants older than 69 years	1/100 (1.0%)
Age-eligible sensitivity N (40-69 years)	54

Supplementary Table S8. Sensitivity analyses restricted to participants aged 40-69 years.

Analysis	N	Effect estimate	p	q	Interpretation
Spearman: endpoint SCORE2 vs T/B exhaustion index (age 40-69)	52	-0,045	0,753		No meaningful correlation in the validated SCORE2 age range
Mann–Whitney: smokers vs nonsmokers for SCORE2 endpoint (age 40-69)	54	0,629	0,000	0,000	Smoking comparison in SCORE2 age-eligible subgroup
Mann–Whitney: smokers vs nonsmokers for adaptive_exhaustion_index (age 40-69)	52	0,134	0,424	0,565	Smoking comparison in SCORE2 age-eligible subgroup
Mann–Whitney: smokers vs nonsmokers for immune_exhaustion_index (age 40-69)	47	0,302	0,094	0,189	Smoking comparison in SCORE2 age-eligible subgroup
Mann–Whitney: smokers vs nonsmokers for second.Frequency of lymphocytes Th(CD4+) PD-1 [%] (age 40-69)	52	-0,023	0,895	0,895	Smoking comparison in SCORE2 age-eligible subgroup

Supplementary Table S9. Age distribution of the cohort.

Age band	N	Percent
21-29	13	0,130
30-39	32	0,320
40-49	41	0,410
50-59	10	0,100
60-69	3	0,030
70-79	1	0,010

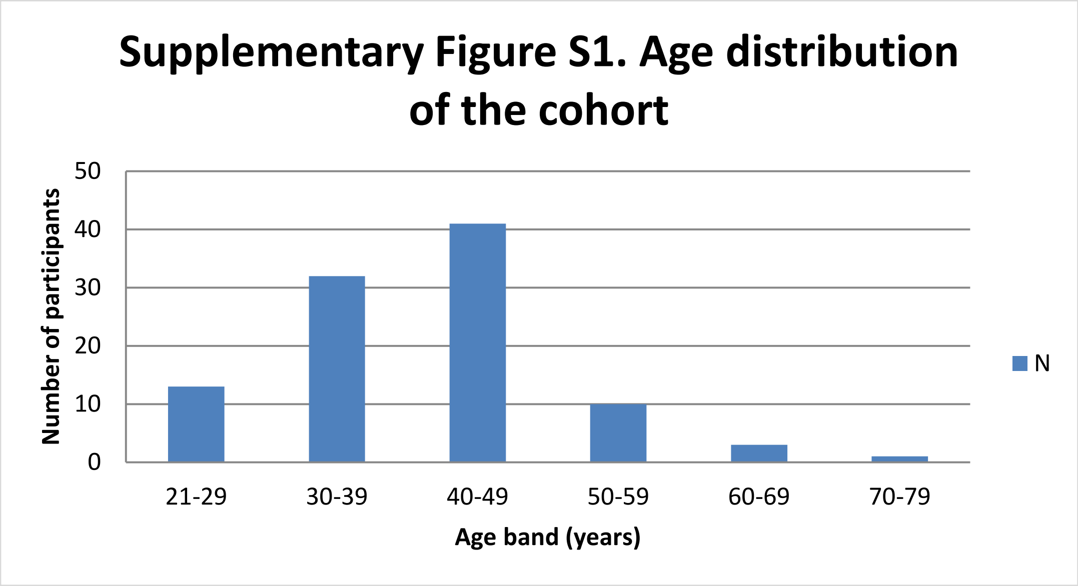

Supplementary Table S10. Primary adjusted smoking models.

outcome_label	n	beta	ci_low	ci_high	p	q	max_cooks	n_cooks_gt_4n	n_high_leverage
T/B-cell exhaustion index	96	-0,225	-0,565	0,115	0,195	0,565	0,140	7	13
Expanded T/B/NK exhaustion index	89	-0,191	-0,562	0,180	0,314	0,565	0,207	5	11
CD3+ T-cell PD-1 endpoint (%)	96	-4,063	-9,269	1,142	0,126	0,565	0,175	7	13
CD3+ T-cell PD-L1 endpoint (%)	96	-0,362	-3,102	2,379	0,796	0,855	0,259	2	13
CD4+ T-cell PD-1 endpoint (%)	96	-5,111	-10,643	0,421	0,070	0,565	0,078	5	13
CD4+ T-cell PD-L1 endpoint (%)	96	-0,395	-2,830	2,041	0,751	0,855	0,434	10	13
B-cell PD-1 endpoint (%)	96	-0,075	-0,885	0,734	0,855	0,855	0,340	3	13
B-cell PD-L1 endpoint (%)	96	-0,283	-0,798	0,232	0,282	0,565	0,181	5	13
NK-cell PD-L1 endpoint (%)	89	-2,077	-17,662	13,508	0,794	0,855	0,190	8	11
